# Studies of post-partum placentas provide insights into the origin of structural chromosomal aberrations

**DOI:** 10.1093/humrep/deaf235

**Published:** 2025-12-03

**Authors:** S H Thomsen, A van Berkel, S van Veen, N van Koetsveld, M Joosten, K E M Diderich, M van den Born, M I Srebniak, D Van Opstal

**Affiliations:** Center for Fetal Diagnostics, Department of Clinical Medicine, Aarhus University, Aarhus N, Denmark; Department of Clinical Genetics, Aarhus University Hospital, Aarhus N, Denmark; Department of Clinical Genetics, Aalborg University Hospital, Aalborg, Denmark; Department of Clinical Genetics, Erasmus MC, University Medical Center, Rotterdam, The Netherlands; Department of Clinical Genetics, Erasmus MC, University Medical Center, Rotterdam, The Netherlands; Department of Clinical Genetics, Erasmus MC, University Medical Center, Rotterdam, The Netherlands; Department of Clinical Genetics, Erasmus MC, University Medical Center, Rotterdam, The Netherlands; Department of Clinical Genetics, Erasmus MC, University Medical Center, Rotterdam, The Netherlands; Department of Clinical Genetics, Erasmus MC, University Medical Center, Rotterdam, The Netherlands; Department of Clinical Genetics, Erasmus MC, University Medical Center, Rotterdam, The Netherlands; Department of Clinical Genetics, Erasmus MC, University Medical Center, Rotterdam, The Netherlands

**Keywords:** structural chromosomal aberrations, placenta, NIPT, prenatal diagnosis, confined placental mosaicism

## Abstract

**STUDY QUESTION:**

Can comprehensive cytogenetic follow-up of the placenta post-partum uncover possible explanations for discrepancies between non-invasive prenatal testing (NIPT) showing structural chromosomal aberrations and foetal follow-up showing normal results or other chromosomal aberrations?

**SUMMARY ANSWER:**

In 18/31 (58%) cases of structural chromosomal aberrations detected with NIPT, where foetal and maternal follow-up was normal or the foetus had another chromosomal aberration, genome-wide examination of term placental chorionic villi confirmed the discrepancy and in 7/18 (39%) confirmed cases complex foeto-placental mosaicism was found.

**WHAT IS KNOWN ALREADY:**

Complex chromosomal rearrangements are often seen in single-cell studies of preimplantation embryos, but it is unknown if these persist into the mature placenta. Confined placental mosaicism explains most discordant NIPTs involving a trisomy, but little is known about structural chromosome aberrations.

**STUDY DESIGN, SIZE, DURATION:**

We performed a retrospective diagnostic test study of cytogenetic follow-up data from post-partum placentas. We included data from pregnancies where (i) NIPT showed a structural aberration, (ii) follow-up of foetus (amniotic fluid and/or cord blood) and mother (genomic DNA and/or cfDNA after birth) was normal or the foetus showed another chromosomal aberration, (iii) follow-up was performed in the Erasmus MC, (iv) more than one sample from the post-partum placenta was analysed, and (v) samples were of good quality (not in formaldehyde, sufficient material).

In the period from January 2014 to March 2022, 115 231 NIPTs were performed in the Erasmus MC; 217 of these showed structural chromosomal aberrations and 123 were followed up in the Erasmus MC (inclusion criteria 3). After exclusion of the foetal (same aberration as with NIPT) and maternal structural chromosome aberrations, 48 placentas were requested to elucidate the discrepancies seen between NIPT (abnormal) and foetal karyotype (normal or differently abnormal; inclusion criteria 1-2). Of these, 31 met criteria 4 and 5 and were included in this study.

**PARTICIPANTS/MATERIALS, SETTING, METHODS:**

In a diagnostic setting, we performed a cytogenetic analysis of postpartum placentas in order to confirm confined placental mosaicism in 31 cases in which NIPT showed a structural chromosome aberration. Two to four chorionic villus biopsies were taken per placenta, and separated enzymatically into cytotrophoblast (CTB) and mesenchymal core (MC) and analysed using SNP arrays. In our analysis, cases were assessed for copy number variants ≥0.5 Mb and regions of homozygosity ≥3 Mb.

**MAIN RESULTS AND THE ROLE OF CHANCE:**

In 18/31 cases (58%), we could confirm the structural chromosome aberration detected with NIPT in one or more placental biopsies. In 13/31 cases (42%), the structural chromosomal aberration detected with NIPT was not confirmed, but in one case an apparently unrelated aberration was found in the CTB of two biopsies. In 11/18 confirmed cases, the same aberration as detected with NIPT, was confirmed in the placenta. All these cases concerned chromosomally normal foetuses with a chromosome aberration confined to the placenta. In one case, an extra, apparently unrelated, aberration was found in one placental biopsy. In 7/18 confirmed cases, the aberration detected with NIPT was confirmed in the placenta and showed to be involved in complex mosaicism involving different abnormal cell lines. In four of these seven cases, the foetus was affected with a pathogenic chromosome aberration that was different from the NIPT aberration. In three cases, a related but benign chromosome aberration was present in the foetus.

**LIMITATIONS, REASONS FOR CAUTION:**

As conventional karyotyping, FISH or whole genome sequencing were not performed, we can only hypothesize on the mechanisms behind the origin of the complex foeto-placental mosaicism we see in seven cases, although there is more strong evidence in the literature as well.

**WIDER IMPLICATIONS OF THE FINDINGS:**

Our results have several implications. First, a genome-wide rather than a targeted approach in foetal follow-up examinations after NIPT showing a structural chromosomal aberration is warranted, as other aberrations may be overlooked in the foetus. Second, counselling of pregnant couples after NIPT showing a structural chromosomal aberration should focus not only on the specific aberration detected by NIPT but also on the possibility that the foetus may harbour another, possibly related, structural aberration. Additionally, a structural chromosomal aberration of apparently uncertain significance may unmask truly pathogenic aberrations in the foetus itself, so all potential foetal cases of structural chromosome aberrations should be followed up. Third, we show how comprehensive examinations of the placenta, specifically the separation of CTB and MC, provides crucial insights into the embryonic origins and mechanisms behind structural chromosomal aberrations. Lastly, our results show that complex chromosomal rearrangements as often seen in single-cell studies of pre-implantation embryos, are not artefacts but a biological phenomenon that can actually persist into the mature placenta and foetus.

**STUDY FUNDING/COMPETING INTEREST(S):**

Simon H. Thomsen was funded by a private research donation and the Department of Clinical Medicine, Aarhus University. All authors declare no conflict of interest.

**TRIAL REGISTRATION NUMBER:**

N/A.

## Introduction

Non-invasive prenatal testing (NIPT) as well as chorionic villus sampling (CVS) for detection of foetal chromosome aberrations rely on examination of placental DNA. In the vast majority, the chromosomal constitution of the placenta and foetus is identical since they both originate from the zygote. In some cases, however, there are discrepancies between the two, termed foeto-placental mosaicism. Studies of chorionic villi (CV) estimate the prevalence of mosaicism to be 2–4% in high-risk pregnancies (Grati *et al.*, 2017; Lund *et al.*, 2020), while studies of NIPT and follow-up investigations in a general obstetric population report a prevalence of approximately 0.2% (van Prooyen Schuurman *et al.*, 2022). Studies of embryos at the cleavage and blastocyst stage estimate that the prevalence of mosaicism at this early point is much higher, if not 100% (Vanneste *et al.*, 2009; Voet *et al.*, 2011; Chavli *et al.*, 2024), but the abnormal cells are then selected against or apparently preferentially allocated to placental lineages in those that develop further (Bolton *et al.*, 2016; Levy *et al.*, 2021). This results in a lower prevalence of mosaicism in CV studies than in studies on cleavage or blastocyst stage embryos and most mosaic aneuploidies being placenta confined (confined placental mosaicism, CPM) after 10 weeks of gestation (Thomsen *et al.*, 2024). Recent studies also show that abnormal cells are unevenly distributed in the placenta (Lund *et al.*, 2021; Eggenhuizen *et al.*, 2024). In contrast to numerical chromosome aberrations, de novo structural chromosomal aberrations (SAs) detected with NIPT or in CV show a markedly higher foetal confirmation rate (Lund *et al.*, 2020; van Prooyen Schuurman *et al.*, 2022). Studies of post-partum placentas have revealed complex mosaic patterns of SAs, most prevalent for terminal chromosome aberrations (Kasak *et al.*, 2015; Van Opstal *et al.*, 2019; Lund *et al.*, 2021; Wang *et al.*, 2025), often even more complex than the original NIPT or CV result.

**Figure 1.**
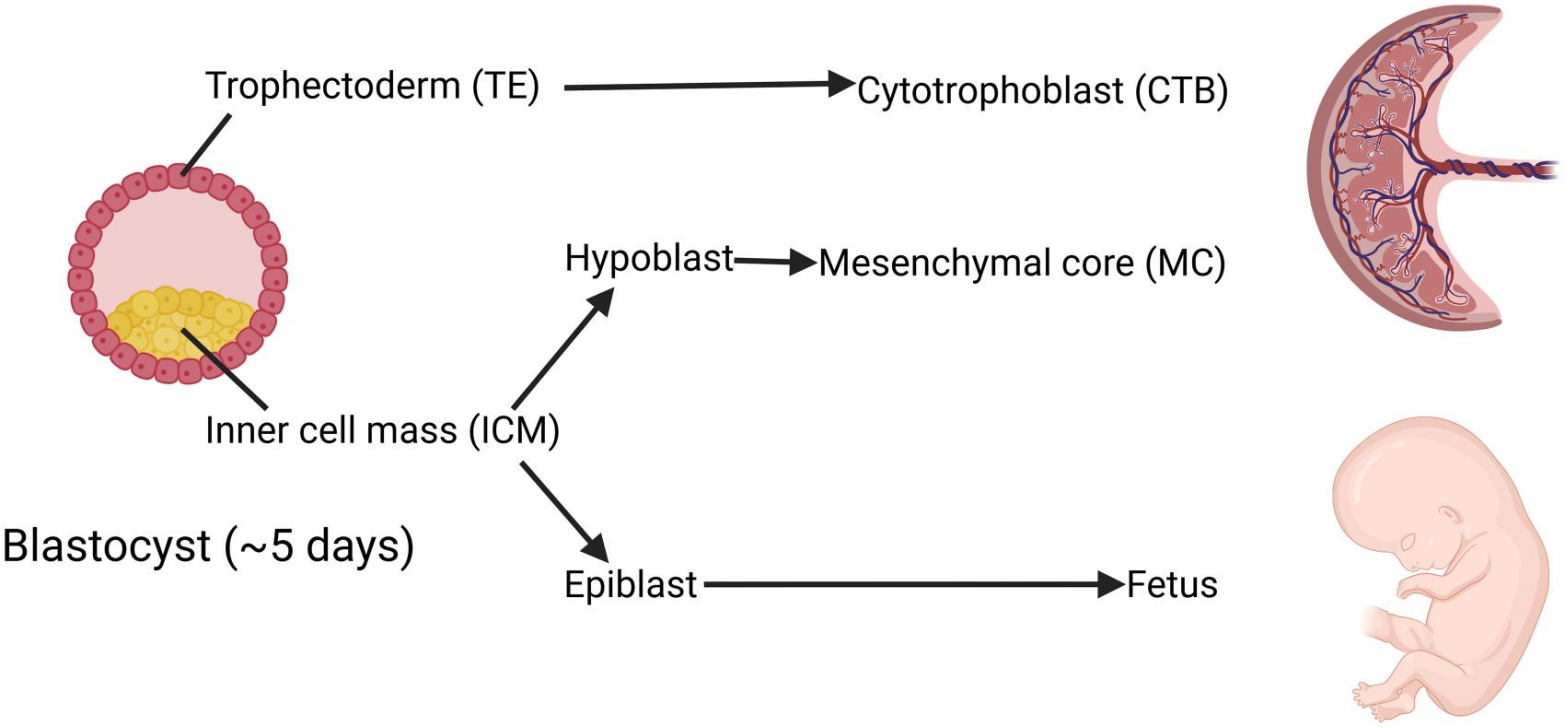
**Blastocyst at ∼5 days gestation and the differentiation of trophectoderm (TE) and inner cell mass (ICM).** The TE forms the cytotrophoblast (CTB) of chorionic villi from which the syncytiotrophoblast is derived, while the ICM differentiates into the hypoblast and the epiblast. The hypoblast forms the extraembryonic mesoderm giving rise the mesenchymal core of chorionic villi. The epiblast forms the foetus. Created in BioRender. Thomsen, S. (2025) https://BioRender.com/z6n3idv.

At the blastocyst stage, cells are already differentiated into the trophectoderm (TE) and inner cell mass (ICM). The TE forms the cytotrophoblast (CTB) and syncytiotrophoblast of CV, while the ICM will further divide into the hypoblast (forming the mesenchymal core (MC) of CV) and the epiblast (forming the foetus) (Eggenhuizen *et al.*, 2021; West and Everett, 2022) (Fig. 1). While NIPT solely investigates DNA from the CTB of CV, examination of CV may involve investigation of both CTB and MC of CV. To investigate the foetus itself, amniocentesis (AC) needs to be performed. We have previously shown how the separate investigation of CTB and MC of placental CV and foetus can reveal different derivatives of a single early event (Van Opstal *et al.*, 2019), as described in cleavage and blastocyst stage embryos (Vanneste *et al.*, 2009; Voet *et al.*, 2011; Chavli *et al.*, 2024). These early events are most probably the cradle of the cytogenetic anomalies seen in term placental studies.

The origins of the complex aberrations seen in embryonic and term placental studies are not fully understood. In a recent paper, Zuffardi *et al.* (2022) hypothesized how terminal deletions could be the result of an isodicentric chromosome whose asymmetric break gives rise to a cell with a simple deletion and another with the reciprocal deletion flanked by an inverted duplication (inv-dup-del). The formation of the isodicentric, often paternal in origin, happens either premeiotically or postzygotically as the result of repair mechanisms of a double-strand break (Hermetz *et al.*, 2014; Kato *et al.*, 2020). The two derivates (del and inv-dup-del) can then be stabilized in various ways: (i) telomere capture of the same arm of the homologue resulting in segmental uniparental disomy (segUPD), or capture of the opposite chromosome arm resulting in a duplication, (ii) telomere capture of another chromosome resulting in an unbalanced translocation (Zuffardi *et al.*, 2022), (iii) telomere healing (Chabchoub *et al.*, 2007) with the formation of a neo-telomere, or (iv) formation of a ring (Zuffardi *et al.*, 2022). Before stabilization, the derivative chromosomes can undergo sequential breakage-fusion-bridge cycles creating even more complex rearrangements (Zuffardi *et al.*, 2022).

In this paper, we investigate (i) whether we can confirm the placental origin of SAs detected with NIPT when follow-up of the foetus did not confirm the NIPT results, (ii) whether new/additional chromosomal aberrations can be detected in the post-partum placenta using a whole genome approach, and (iii) whether the cytogenetic results of the term placenta can explain the cytogenetic differences between NIPT and foetus. Furthermore, we show how a thorough and genome-wide investigation of the CTB and MC of CV can provide important information for counsellors and patients explaining the discrepant results between NIPT and foetus.

## Materials and methods

**Figure 2.**
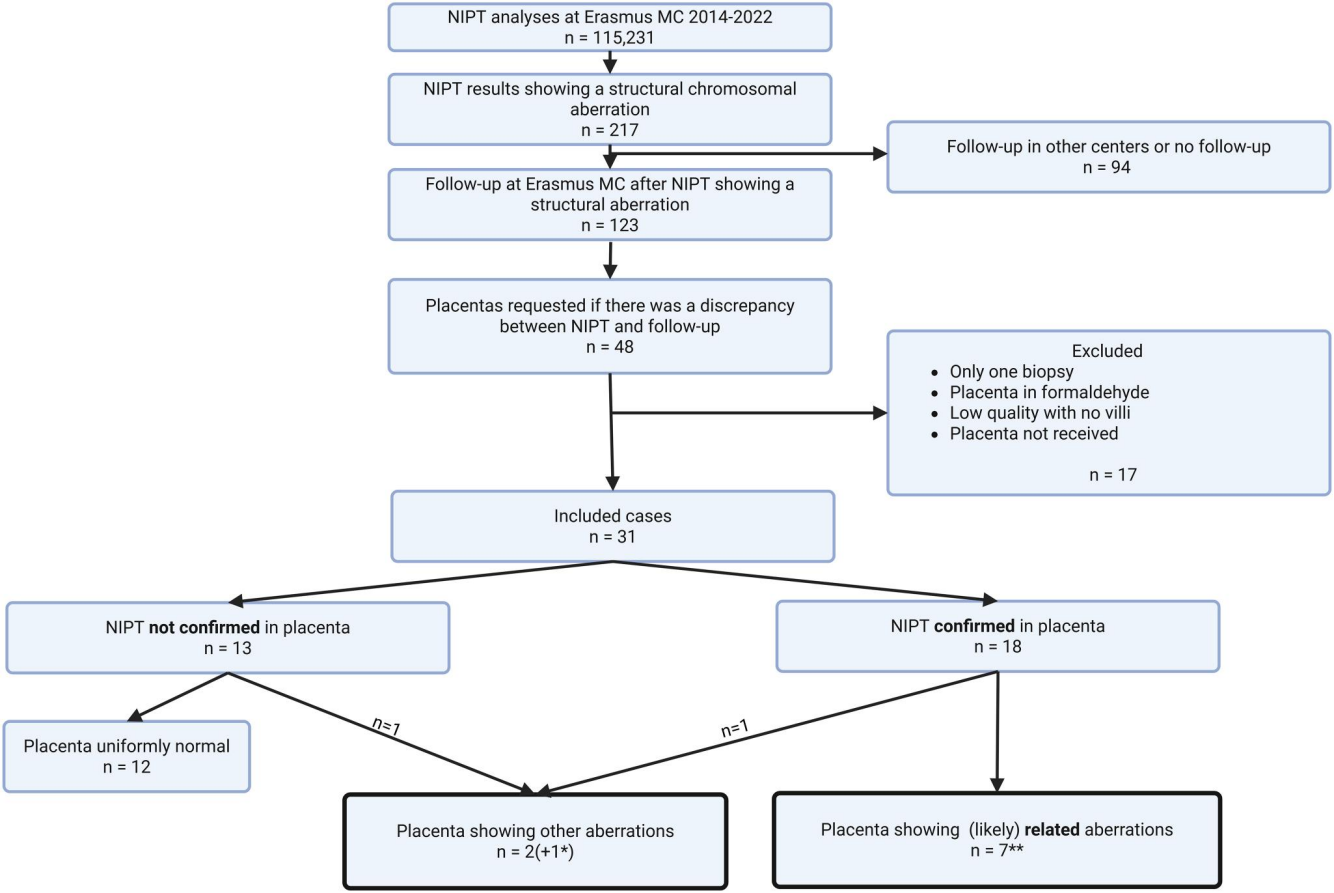
**Flowchart of cases included in this study.** *Case 4 showed likely related aberrations, but in one sample also a likely unrelated mosaic segUPD of 11p. **Three cases previously described (Van Opstal *et al.*, 2019) but included in total numbers here. Created in BioRender. Thomsen, S. (2025) https://BioRender.com/48ekdow.

In the period from April 2014 to March 2022, 115 231 NIPTs were performed at the Erasmus MC, Rotterdam, The Netherlands (Fig. 2). In 217/115 231 (0.19%) cases, NIPT showed SAs. In case of an abnormal NIPT result, follow-up testing was offered. If the results of amniotic fluid or chorionic villi were discrepant from the NIPT result, cytogenetic investigation of the post-partum placenta (four biopsies from each quadrant of the placenta) was requested in all cases where follow-up investigations were done in the Erasmus MC. Cases that showed concordant abnormal results in the prenatal follow-up or a maternal origin of the NIPT aberration (due to maternal germline CNVs or the presence of leiomyoma) were excluded.

In the study period, we requested 48 placentas for analysis in diagnostic settings. Data of 31/48 post-partum placentas with two to four biopsies of good quality are included in this paper. In 17/48 cases, only one biopsy was sent (which did not allow assessing mosaicism within the placenta) or placentas/placental biopsies were not of good quality (e.g. fixed in formaldehyde, not enough material for enzymatic separation) or biopsies were not received. Three of these 31 cases (cases 1-3) have been described in detail previously (Van Opstal *et al.*, 2019), but are included in the total numbers of the cohort. One case, case 7, was included in a previous paper with a different scope (Donze *et al.*, 2024), so not described in detail; therefore, it is described here.

Fresh placentas were biopsied on the maternal side and CTB and MC of the CV were enzymatically separated according to standard protocols (Van Opstal *et al.*, 2018). Briefly, the CTB was removed with trypsin and after sieving it (to remove small pieces of MC) DNA was isolated from the filtrate. The MC was digested with collagenase before DNA isolation. DNA from both cell lineages was analysed with SNP array (Infinium-CytoSNP-850K genotyping array or Infinium GSA + MD-24 v1.0 BeadChip, Illumina, USA). Data analysis was done with Nexus Copy Number (Bionano Genomics, USA). All samples were evaluated for copy number variants (CNVs) ≥0.5 Mb and regions of homozygosity (ROH) >3 Mb across the entire genome. Smaller CNVs down to 0.1 Mb were recorded when in relation to larger SAs or to the SA initially detected with NIPT. Aberrations detected on the same chromosome as seen with NIPT or aberrations involving a terminal gain of another chromosome, potentially involved in a translocation, were considered potentially related to the initial SA detected with NIPT, according to Zuffardi *et al.* (2022). All other chromosomal aberrations were considered likely unrelated.

Blood of both parents was routinely collected and analysed with the same SNP-array, and SNPs were used to determine on which parental chromosome the aberrations occurred. In mosaic cases, the mitotic or meiotic origin of a gain was determined by the B-allele frequency plot (BAF) as described by Conlin *et al.* (2010). If only normal or non-mosaic samples were available, the origin could be determined making digital mosaics as previously described (Van Opstal *et al.*, 2018).

### Ethical approval

Here, we describe the cytogenetic follow-up in foetuses with high-risk NIPT results identified in the TRIDENT-1 study and the TRIDENT-2 study in 2014-2022. Permission for the TRIDENT-1 study was granted by the Dutch Minister of Health, Welfare and Sport (350010-118701-PG) on March 28, 2014. A license for the TRIDENT-2 study was granted by the Dutch Minister of Health, Welfare and Sport (1017420-153371-PG) on September 20, 2016. Additionally, the study in the general population was approved by the Medical Ethics Committee of the Amsterdam UMC, VU University Medical Center (VUMC No.2017.165) on 27 March 2017. All women consented to their data being used for research purposes.

## Results

**Figure 3.**
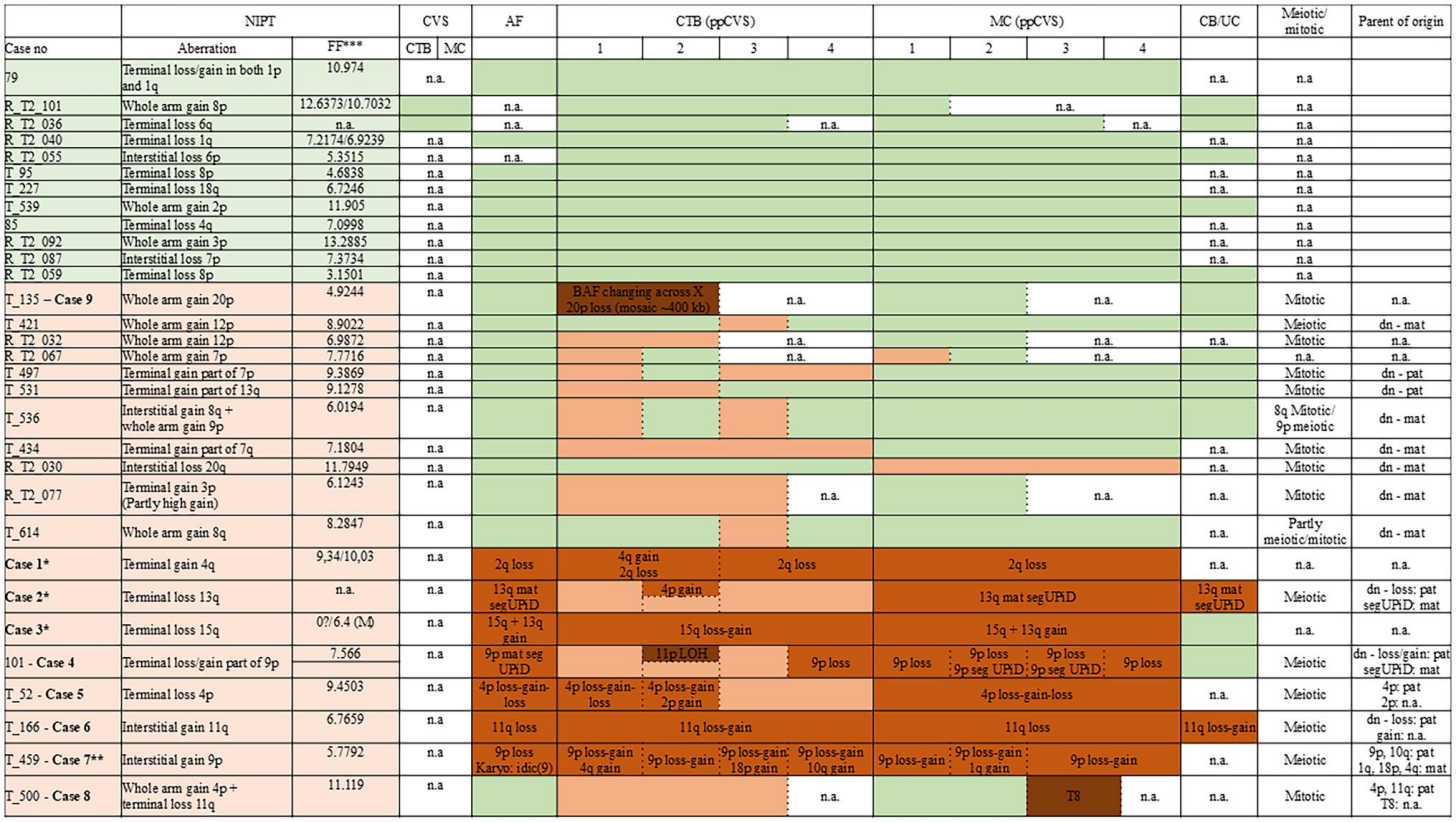
**Overview of included cases.** Cases in bold (cases 1-9) are cases with additional findings, and described in detail. Case numbers in light green: Post-partum placenta uniformly normal (12 cases). Case numbers in light orange: NIPT confirmed (18 cases) and/or additional aberrations (1 case, case 9). Green indicates normal array findings, light orange indicates exactly the same aberration as with NIPT, medium brown indicates aberrations probably related to the NIPT result, and dark brown indicates aberrations probably unrelated to the NIPT result. NIPT, non-invasive prenatal test; CVS, chorionic villus sampling (during pregnancy); CTB, cytotrophoblast of chorionic villi; MC, mesenchymal core of chorionic villi; AF, amniotic fluid; ppCVS, post-partum chorionic villus sample; CB/UC, cord blood/umbilical cord; dn, de novo; mat/pat, maternal/paternal; LOH, loss of heterozygosity. Meiotic or mitotic origin based on B-allele frequencies. Parent of origin determined based on SNP analysis of foetus and both parents. *Cases described previously in Van Opstal *et al.* (2019). **Case briefly included in Donze *et al.* (2024). ***Foetal fraction. SeqFF where available and otherwise DEFRAG SY/WY (Hestand *et al*., 2019).

In our cohort of 31 post-partum placentas from pregnancies with potential CPM for a SA detected with NIPT, or with a foetal SA that differed from the NIPT, the aberration as detected with NIPT was confirmed in the placenta in 18 cases (18/31, 58%) (Figs 2 and 3).

In 11/18 (61%) cases, foetal chromosome studies were normal and the same aberration as detected with NIPT was found in the placenta (9 only in CTB, 1 only in MC, and 1 in both). In one case, case 8, an additional, probably unrelated, trisomy 8 was detected in MC of one biopsy.

In 7/18 cases (39%), complex mosaicism was found with a different chromosome aberration than detected with NIPT present in the foetus (Table 1). Placental investigations allowed clarification of the discrepancy between foetus and NIPT. Apart from the NIPT aberration, all cases showed additional anomalies, which were probably related to the NIPT result, except for the mosaic ROH on 11p in case 4, and the placenta showed more complexity (gain/loss/ROH). In 4/7 cases, the foetal SA was pathogenic (cases 1, 3, 5, and 6). In the two cases with maternal segUPiD (cases 2 and 4), no homozygous pathogenic variants of maternal origin were detected with targeted whole exome sequencing. The ∼200 Kb loss on 9p in the foetus of case 7 was benign.

**Table 1.** Cases of complex foeto-placental mosaicism: NIPT result, amniocentesis results, and pregnancy outcome.

Case	NIPT	Fetal results (amniotic fluid)	Pregnancy outcome
1	Terminal gain 4q*	SNP-array: ∼10 Mb loss of 2q37.1q37.3 arr[hg19] 2q37.1q37.3(232,717,857_243,048,760)x1dn Karyotype: 46,XY,del(2)(q37.1)	Pregnancy terminated 2q37 deletion syndrome
2	Terminal loss 13q*	SNP array: maternal segmental uniparental isodisomy of 13q31.3q34 arr[hg19] 13q31.3q34(90252671_115103529)x2 hmz mat No homozygosity for a pathogenic variant in an autosomal recessive gene on 13q	Healthy girl born at 38 weeks and 1 day of gestation (birth weight 3120 g). No congenital malformations and normal development at 2 years of age.
3	Terminal loss 15q*	SNP array: mosaic ∼36 Mb gain of 15q22.31q26.3 and ∼10 Mb gain of 13q33.2q34 (5-10%) arr[hg19] 15q22.31qter(66,612,725_102,461,162)x2∼3, 13q33.2qter(105,015,223_115,103,529)x2∼3 dn Karyotype: 46,XY,der(13)t(13;15)(q34;q22.31)/46,XY	Results confirmed in buccal mucosa after birth Developmental delay at 8 years of age.
4	Terminal loss/gain 9p	SNP array: maternal segmental uniparental isodisomy of 9p24.3p24.1 arr[hg19] 9p24.3p24.1(133,828_7,497,932)x2 hmz mat No homozygosity for a pathogenic variant in an autosomal recessive gene on 9p	Healthy child born at 41 weeks and 1 day of gestation. Healthy at 2 years of age.
5	Terminal loss 4p	SNP array: four interstitial losses and one gain of 4p16.3p16.1 arr[hg19] 4p16.3(582,067_1,312,043)x1,4p16.3(∼1,700,000_1,900,000)x3,4p16.3p16.1(1,929,117_6,489,251)x1,4p16.1(∼6,550,000-7,000,000)x1 dn	Pregnancy terminated Wolf Hirschhorn syndrome
6	Interstitial gain 11q	SNP array: ∼9 Mb loss of 11q24.2q25 arr[hg19] 11q24.2q25(125,864,878_134,945,120)x1 dn	Pregnancy terminated Jacobsen syndrome
7	Interstitial gain 9p**	SNP array: ∼200 Kb loss of 9p24.3 arr[hg19] 9p24.3(133,828_326,767)x1 dn Karyotype: 46,XX,idic(9)[1]/46,XX[36]	Pregnancy continued Healthy child born at 39 weeks and 6 days of gestation (birth weight 2464 g). Carriership of autosomal recessive condition involving the *DOCK8* gene (Hyper-IgE recurrent infection syndrome, OMIM #243700).

Fetal aberrations in cases 1, 3, 5, and 6 were pathogenic, while cases 2, 4, and 7 had normal pregnancy outcomes.

* Cases described previously in Van Opstal *et al.* (2019).

** Case briefly included in Donze *et al.* (2024).

In 13 cases of the total cohort (13/31, 42%), the SA detected with NIPT was not confirmed in the placenta. Twelve of these 13 cases showed uniformly normal results at a resolution of 0.5 Mb, while one showed other chromosomal aberrations, not detected with NIPT (case 9).

### Cases with additional chromosome aberrations

Thus, in a total of nine cases (9/31, 29%), additional SAs were detected in the term placenta using a whole genome approach (Fig. 3). In two of these, the aberrations were completely different and seemingly unrelated to the NIPT result (cases 8 and 9), while in the other seven (cases 1-7), the aberrations were likely related. Case 4, also showed a likely unrelated mosaic ROH on 11p, apart from the related aberrations on 9p.

Separation of CTB and MC revealed that MC more often resembled the chromosomal constitution of the foetus; however, in some cases, additional aberrations were present in the MC as well (cases 4, 7, and 8). In contrast, CTB resembled the NIPT result, however, often with additional aberrations. Comparing CTB and MC, they often showed different, but likely related, chromosomal constitutions with CTB showing more complex aberrations and differences between CTB samples from the same placenta, while the MC was less complex and most often showed the same aberration in all MC samples.

All 9 cases with additional aberrations are shown in Fig. 3 (cases 1-9). For details on cases 1-3, see our previous paper (Van Opstal *et al.*, 2019). Briefly, in case 1, NIPT showed a gain on 4q, while the foetus had a terminal loss on 2q. The 4q gain was confirmed in CTB of the post-partum placenta, while the 2q loss was confirmed in both CTB and MC, although with different lengths. In case 2, NIPT showed a terminal loss of 13q, while the foetus had maternal segUPiD of the same segment. The 13q loss was confirmed in CTB, in different lengths, along with a gain on 4p in one biopsy, while the MC showed the same segUPiD of 13q as the foetus. In case 3, NIPT showed a loss of 15 q, but in the foetus a gain on 13q and 15q was detected. The 15q loss was confirmed in CTB, along with the 15q gain, while the MC resembled the 13q and 15q gains in the foetus.

**Figure 4.**
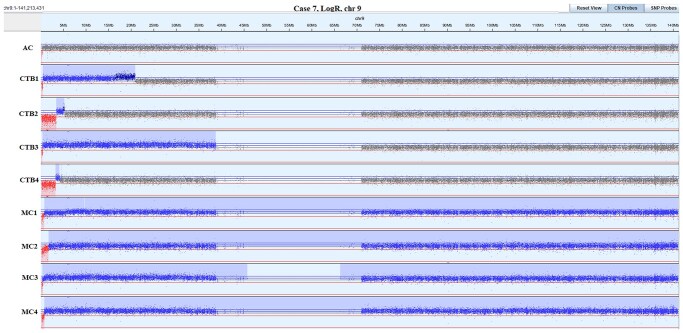
**LogR plot of chromosome 9 in amniotic fluid (AF), cytotrophoblast biopsies 1-4 (CTB1-4) and mesenchymal core biopsies 1-4 (MC1-4) in case 7 with NIPT showing an interstitial gain on 9p (see Supplementary Fig. S1).** Follow-up analysis of AF showed a small (0.2 Mb) terminal loss on 9p. The gain was confirmed in all CTB and MC samples, and differed in length ranging from ∼2 Mb and up to 140 Mb in all MC samples (spanning the entire chromosome 9, except the terminal loss). All biopsies also showed a terminal loss of 9p as in the amniotic fluid. These losses were also of different length ranging between 0.2 Mb and ∼3 Mb in the CTB fractions, whereas mosaicism for different length deletions was seen in each MC sample. All these chromosome 9 aberrations occurred on the paternal allele. Several additional aberrations were also detected (see Supplementary Fig. S1): in CTB1, a 97 Mb terminal gain of the maternal 4q; in CTB3, a 2.4 Mb terminal gain of the maternal 18p; in CTB4, a 82 Mb terminal gain of the paternal 10q; and in MC2, a 15 Mb gain of the maternal 1q. Karyotyping of amniotic fluid showed an isodicentric chromosome 9 in 1/37 cell colonies.

The six other cases, which have not been previously described (cases 4-9) are summarized below. Details can be found in Supplementary Table S1. SNP-array plots of these cases are exemplified in Fig. 4 (case 7) and Supplementary Fig. S1 (cases 4-9).

In four of the six cases not previously described, the additional aberrations were likely related to the NIPT result (cases 4-7):

- In case 4, NIPT showed a profile suggestive for a derivative chromosome 9 with a terminal deletion of 9p with coexisting interstitial 9p gain, while a maternal segUPD of the fragment that was lost was found in the foetus (amniotic fluid; AF). A healthy child was born at 41 weeks and 1 days of gestation. The NIPT result was confirmed in 3 of 4 CTB samples (CTB 1-3); however, the loss and gain had different lengths in different CTB samples. In CTB 4 only, the loss was confirmed, which was also the case in all MC samples. The loss in CTB 4 was, however, longer than the loss detected in MC and segUPD in AF, which were almost identical in length. All these events occurred on the paternal allele. In two MC samples (MC 2-3), SNP array showed a mosaic loss coexisting with a maternal segUPD in the other cell line. As the foetal DNA only showed segUPD in 9p, targeted exome sequencing was used to exclude pathogenic variants in recessive genes on 9p in the foetus. Furthermore, in CTB 2, a mosaic ROH on 11p was detected. At the age of 2 years, the child is healthy.
- In case 5, NIPT showed an interstitial loss on 4p; however, in the foetus (AF) a different pattern showing consecutive interstitial losses and an interstitial gain on 4p, but with normal copy number and biparental contribution at the very terminal end was seen. This chromosome aberration was associated with Wolf-Hirschhorn syndrome (Battaglia *et al.*, 2015). The pregnancy was terminated at 18 weeks and 1 day of gestation. All CTB samples showed consecutive losses with small areas with normal copy number in between. These losses were followed by a gain of different lengths in two CTB samples (CTB 1-2). Furthermore, in CTB 2, a mosaic 15 Mb terminal gain on 2p was also detected. All MC samples resembled the aberrations in the foetus. The aberrations on chromosome 4 occurred on the paternal allele, while the parental origin of the 2p gain could not be determined due to maternal cell contamination.
- In case 6, NIPT showed an interstitial gain on 11q while the foetus (AF) showed a terminal loss on 11q associated with Jacobsen Syndrome (Mattina *et al.*, 2009). The pregnancy was terminated. The NIPT result was confirmed in all CTB samples, as well as in one MC sample (MC 4), with involved segments of different lengths. Flanking the gain was a terminal loss in all CTB and one MC, while only the loss was detected in the other three MC samples as well as in a sample from the umbilical cord. The losses detected in MC and foetus were of the same length (9.1 Mb), while the loss detected in CTB samples was ∼0.5 Mb longer, but still under the detection limit of NIPT. All losses and gains of 11q originated on the paternal allele.
- In case 7 (Fig. 4), similarly to case 6, NIPT showed an interstitial gain on 9p while the foetus had a small (0.2 Mb) terminal loss on 9p. The small loss resulted in the child being a healthy carrier for an autosomal recessive condition related to the *DOCK8* gene (Donze *et al.*, 2024). A small but healthy child was born at 39 weeks and 6 days of gestation (birth weight 2464 g). The gain was confirmed in all CTB and MC samples, but all samples also showed a terminal loss on 9p as in the foetus. In most samples, the loss was small, too small to be detected with NIPT (∼0.2-3 Mb). The gains, present in each sample (CTB and MC), ranged from ∼1 Mb up to 140 Mb in the MC samples (spanning the entire chromosome 9, except the terminal loss). All aberrations on chromosome 9 were determined to be on the paternal allele. In both CTB and MC samples, several additional aberrations were also detected: in CTB 1 a 97 Mb terminal gain of the maternal 4q, in CTB 3 a 2.4 Mb terminal gain of the maternal 18p, in CTB 4 a 82 Mb terminal gain of the paternal 10q, and in MC2 a 15 Mb gain of the maternal 1q. Karyotyping of AF showed an isodicentric chromosome 9 in 1/37 cell colonies.

In two cases, the additional findings were likely unrelated to the NIPT result (cases 8 and 9):

- In case 8, NIPT showed a gain of the whole 4p and a terminal loss on 11q while the foetus was chromosomally normal. A healthy girl was born at the 39 weeks and 5 days of gestation. The NIPT result was confirmed in all CTB samples and both aberrations involved the paternal alleles. MC was normal except for MC 3 that showed a mosaic trisomy 8.
- In case 9, NIPT showed a gain of the entire 20p, which was not confirmed in the foetus (AF and cord blood) or placenta. UPD 20 was also excluded. A healthy child was born at 39 weeks and 3 days of gestation (birth weight 3450 g). In both CTB samples, results indicated a complex result for the X chromosome, where the BAF showed a very complex pattern through the entire X chromosome, but with normal copy number. Furthermore, a mosaic small (∼400 Kb) interstitial loss on 20p was detected in both CTB.

## Discussion

### Main findings

Using a whole genome approach on enzymatically separated chorionic villi, we confirmed SAs detected with NIPT with suspected placental origin, in 58% (18/31) of post-partum placentas. In 11/18 cases in which the foetus was cytogenetically normal, exactly the same chromosome aberration as detected with NIPT was shown in the placenta. In 7/18 cases in which the foetus showed a different chromosome aberration than expected based on the NIPT result, placental studies revealed the NIPT aberration as well as additional chromosome aberrations. In these cases, the aberration detected with NIPT was found to be part of a much more complex foeto-placental mosaicism involving additional most probably mostly related chromosomal aberrations and differences between CTB and MC. These differences likely explained the discrepancies between NIPT and follow-up cytogenetic investigations of the foetus. In addition to these 7 cases, there were two other placentas (cases 8 and 9) with most probably unrelated chromosome aberrations in a few biopsies showing an increased complexity in the placenta in a total of 29% (9/31) of the cases.

The separation of CTB and MC and the finding of different SAs in these two cell lineages along with results from NIPT and foetus allows us to make qualified hypotheses on the origins of these aberrations.

### Possible mechanisms behind complex foeto-placental mosaicism

Zuffardi *et al.* (2022) hypothesize that terminal deletions are the result of an original isodicentric chromosome that may break during cell division leading to simple deletions and their reciprocal inv-dup-del derivative chromosomes. At least four of our cases support this hypothesis, where we see cell lines with terminal losses and terminal losses flanked by gains in the post-partum placenta (cases 3, 4, 6, and 7). In two of these (cases 6 and 7), only the loss was present in the foetus. Here the post-partum placenta gave a unique insight into the different cell lines arising from the hypothesized isodicentric chromosome. In case 7, there may be cells that even retained the original isodicentric chromosome: in all four MC biopsies, there was a mosaic gain of the entire paternal chromosome 9 flanking the small terminal loss. This is further supported by the finding of an isodicentric chromosome 9 in one cell colony in cultured AF. At first sight, this is in contrast with the above hypothesis, since an isodicentric, having two centromeres, may be pulled to opposite spindle poles and thus break during cell division. However, in these cells one of the two centromeres could be inactivated (Stimpson *et al.*, 2010), allowing occasionally the retention of the isodicentric into the cell.

Terminal deletions are unstable as they lack telomeres. Until stabilized, chromosomes can undergo further several breakage-fusion-bridge cycles, creating complex rearrangements (Zuffardi *et al.*, 2022). This hypothesis is supported by the several cases presented in this cohort showing losses and gains of different lengths and in different proportions of cells (cases 1-2 and 4-7).

These unstable chromosomes can be stabilized in several ways, where Zuffardi *et al.* (2022) hypothesizes three possibilities: telomere capture, telomere healing, and/or ring formation. As we have not performed conventional karyotyping, ring formation could not be investigated, but we see indications of both telomere capture and telomere healing. Telomere capture of the homologue is likely to have occurred in cases 2 and 4. Here we see a terminal loss on the paternal allele, and in the foetus and MC cells maternal segUPiD corresponding to the lost segment, indicating stabilization by telomere capture of the maternal homologue. Telomere capture of the terminal end of another chromosome could have been the stabilizing event of the terminal loss in cases 1, 2, and 7, as we see terminal gains on other chromosomes. In case 7, this even involves four different chromosomes in three different CTB samples and one MC sample. To prove this hypothesis, long-read sequencing would be helpful to confirm that these gains indeed repaired the deleted segments.

Telomere healing with the formation of a neo-telomere could be the stabilizing event in the cell lines where there is a terminal loss and no corresponding gain of the terminal end of another chromosome or mosaic segUPD indicating telomere capture. We see this in some of the samples from cases 1 to 7. Alternatively, ring formation could also have occurred in these cases but was not investigated.

It has been hypothesized that the isodicentrics originate in paternal meiosis (Belyeu *et al.*, 2021; Zuffardi *et al.*, 2022) and our data reflect this. Where we could investigate this, in cases 2 and 4-7, all losses were on the paternal allele, and in cases with gains flanking losses (cases 4, 5, and 7), these were also on the paternal allele. Moreover, in all seven cases of complex foeto-placental mosaicism, all CTB and MC samples were chromosomally abnormal. Altogether this indicates that the original chromosome aberration probably occurred in paternal meiosis.

In case 5, aberrations are not terminal, but rather losses interspersed with copy-neutral regions and gains, with even in one CTB sample a gain of the terminal end of 2p. Here, a likely mechanism could be either chromothripsis, indicated by the consecutive losses, or chromoanasynthesis, indicated by consecutive losses *and* gains. Chromothripsis could also be part of the explanation in case 9 (Burssed *et al.*, 2022).

### Differences between inner cell mass and trophectoderm

In our cases with complex mosaicism, the aberrations in the TE-derived CTB were often more complex while the ICM-derived MC was more stable. As shown by Coorens *et al.* (2021), early developmental “bottlenecks” exist separating the TE from the ICM within the first few cell divisions, and they hypothesize that mechanisms that protect the genome do not operate in the same way in trophoblasts as they do in other tissues. This could explain the higher complexity in CTB; however, it is also possible that this is merely due to the fact that the vast majority of cells in the early embryo will end up in the TE and only a small minority in the ICM, thus making it more likely to have several different cell lines present in the TE. A study by Chavli *et al.* of single cells from TE and ICM at the blastocyst stage showed a similar pattern of related chromosome aberrations and losses and gains of different length within different cells of the same blastocyst, also with a higher incidence of complex chromosomal rearrangements in the TE (Chavli *et al.*, 2024). Our results add to the understanding that these different abnormal cell lines, frequently seen in human blastocysts, are not artefacts but a biological phenomenon that may persist into the mature placenta, probably depending on their stability.

### Clinical implications

CPM has been labelled the major cause of discrepancies between NIPT and foetus (Hartwig *et al.*, 2017). This is indeed the case for the autosomal trisomies as shown in the TRIDENT-2 study where the majority of rare autosomal trisomies were (assumed) CPM (van Prooyen Schuurman *et al.*, 2022). For SAs detected with NIPT, however, there are more things to consider than CPM. The aberration detected by NIPT may be of maternal origin, be present in the foetus, confined to the placenta or the foetus may have a different aberration than detected by NIPT.

We could not confirm a placental origin in 13 (42%) of our cases, which is higher than compared to trisomies (27% (Eggenhuizen *et al.*, 2024)). It is most likely that they were of placental origin, but due to the patchy nature (Eggenhuizen *et al.*, 2024) of the placenta we did not “catch” the aberration with the four biopsies. It is also not completely excluded that they had a maternal origin despite normal SNP array results on maternal blood as well as normal cfDNA testing after birth in most cases: a somatic aberration in a myoma for instance could be detected with NIPT during pregnancy, whereas it may remain undetectable with cfDNA testing after birth (own unpublished data). Lastly, a vanishing twin could also be the explanation for not confirming NIPT results in some of the cases.

As we show here, SAs detected with NIPT, may be representative of a much more complex chromosomal constitution throughout the foeto-placental unit, where the specific aberration detected with NIPT is indeed placenta confined, but *another* aberration is present in the foetus. Furthermore, the NIPT result may not even be fully representative of the chromosomal constitution of the placenta itself. This has already been reported before (Van Opstal *et al.*, 2019), and the current cases further support the previous findings. Our data show that SAs detected by NIPT are probably more often mosaic than what would be expected from cytogenetic studies in AF or blood showing only one cell line. This must be kept in mind when counselling pregnant couples. Counselling should not focus specifically on the aberration seen with NIPT but also on the possibility of the foetus harbouring another, possibly related, SA. Specifically, when detecting a terminal loss/gain or an interstitial, but terminally located gain with NIPT, it should be kept in mind that this could represent a simple loss in the foetus. Moreover, when performing follow-up cytogenetic investigations in CV, AF, or other foetal tissues, this should always be done using high resolution genome-wide and not targeted techniques and preferably by SNP array enabling the identification of a ROH or a submicroscopic loss/gain. In a previous paper, it was recommended to consider performing CVS instead of AF when NIPT showed specific trisomies as the MC would provide a definitive diagnosis in almost all cases (Van Opstal and Srebniak, 2016). However, we do not recommend this when NIPT shows a SA, since the MC did not fully reflect the foetus in 6/31 (19%) cases (case 4, 6-8 + 2 cases where the MC confirmed the NIPT result but the foetus was normal, see Fig. 3). Although a normal MC always correctly reflected the foetal karyotype in the present study, we show here that if a SA is present, it, more often than one may expect, is involved in chromosomal mosaicism and if mosaicism is detected in the MC, the karyotype in the foetus is still unknown and follow-up investigations in AF will be necessary for a definitive prenatal diagnosis. To prevent two invasive tests and a very long waiting-time for the prospective parents, we recommend amniocentesis as the most proper follow-up test.

### Strengths and limitations

The major strength of this study is the comprehensive assessment of cases of NIPT showing SAs and discrepant results in follow-up. The genome-wide assessment of both foetus and post-partum placenta allowed us to not only confirm the placental origin of NIPT results in 58% of cases, but the separation of CTB and MC also allowed us to understand the discrepancies between the NIPT result and the foetal chromosomal constitution. We further gained a unique insight into the early embryonic origins and mechanisms behind SAs.

Unfortunately, as we did not perform conventional cytogenetic analyses such as karyotyping/FISH, except in case 7, we can only hypothesize on the origins and mechanisms of the complex chromosomal aberrations we detected. This will have to be confirmed by further analyses with conventional karyotyping, FISH or whole genome sequencing or optical genome mapping that could potentially provide us with the exact chromosomal architecture of these cases.

## Conclusions

We showed that whole genome placental analyses are valuable for studying mechanisms leading to chromosomal aberrations, but are also important for clinical explanation in cases of discrepant results between NIPT and foetus. We have encountered cases where the foetus carried a pathogenic aberration, different from the one detected by NIPT, which is often difficult to comprehend for the parents. Based on the placental cytogenetic studies the clinical geneticist could explain and show them that the discrepancy had a biological origin. When NIPT indicates a structural aberration, follow-up should be genome-wide (chromosomal microarray or whole genome sequencing with CNV analysis) and performed on AF. Targeted confirmatory studies may miss other aberrations and the mesenchymal core of CV may not be fully representative of the foetus. Counselling should include the possibility of other, possibly related, foetal aberrations in addition to the one(s) detected with NIPT as well as the possibility of CPM or a maternal origin of the NIPT aberration(s).

The comprehensive assessment of NIPT, foetus, and cytotrophoblast and mesenchymal core of CV from the post-partum placenta allowed us to study the mechanisms of how chromosomal rearrangements originate in the early embryo. Importantly, our results add to the understanding that complex chromosomal rearrangements, often seen in human blastocysts, are a biological phenomenon that can persist into the mature placenta and foetus. Preferably long read sequencing or optical genome mapping could be employed in future studies to detect the individual cell lines showing various telomeric captures and other repair events and show the true cytogenomic composition of the aberrant chromosomes.

## Supplementary Material

deaf235_Supplementary_Figure_S1

deaf235_Supplementary_Table_1

## Data Availability

The data underlying this paper cannot be shared in full publicly due to the privacy of individuals participating in the study. Data can be shared in anonymized form on reasonable request to the corresponding author.
