# Supplementary material for: Studies of post-partum placentas provide insights into the origin of structural chromosomal aberrations
Source: Hum Reprod. 2025 Dec 3;41(2):168–77. doi: 10.1093/humrep/deaf235 (PMC12864147; doi:10.1093/humrep/deaf235)
Supplement: deaf235_Supplementary_Figure_S1 [file deaf235_supplementary_figure_s1.pdf]

Supplementary Figure S1

NIPT, LogR and BAF plots of cases 4-9

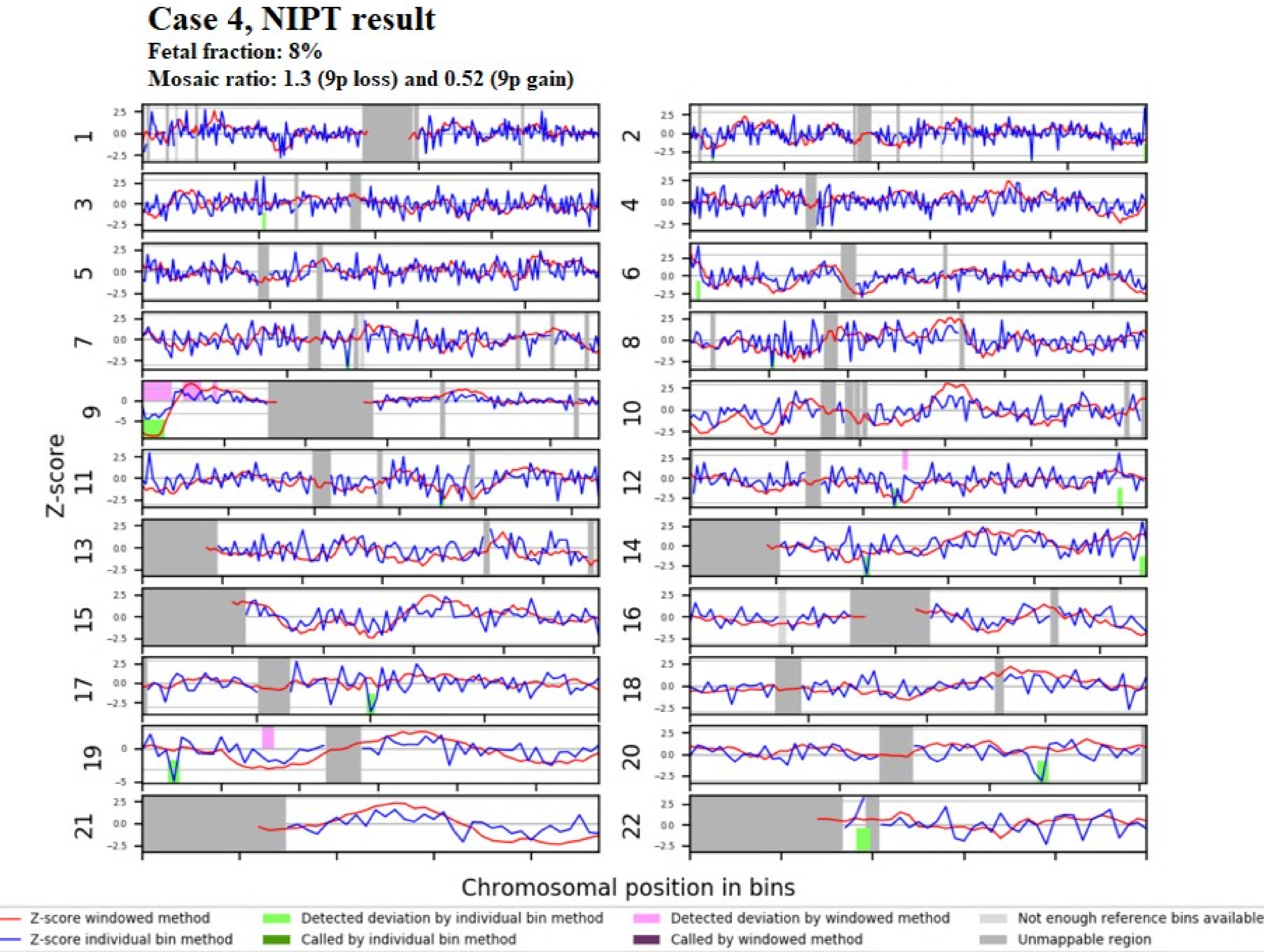
Case 4, NIPT result
Fetal fraction: 8%
Mosaic ratio: 1.3 (9p loss) and 0.52 (9p gain)
Z-score
Chromosomal position in bins
Z-score windowed method
Z-score individual bin method
Detected deviation by individual bin method
Called by individual bin method
Detected deviation by windowed method
Called by windowed method
Not enough reference bins available
Unmappable region

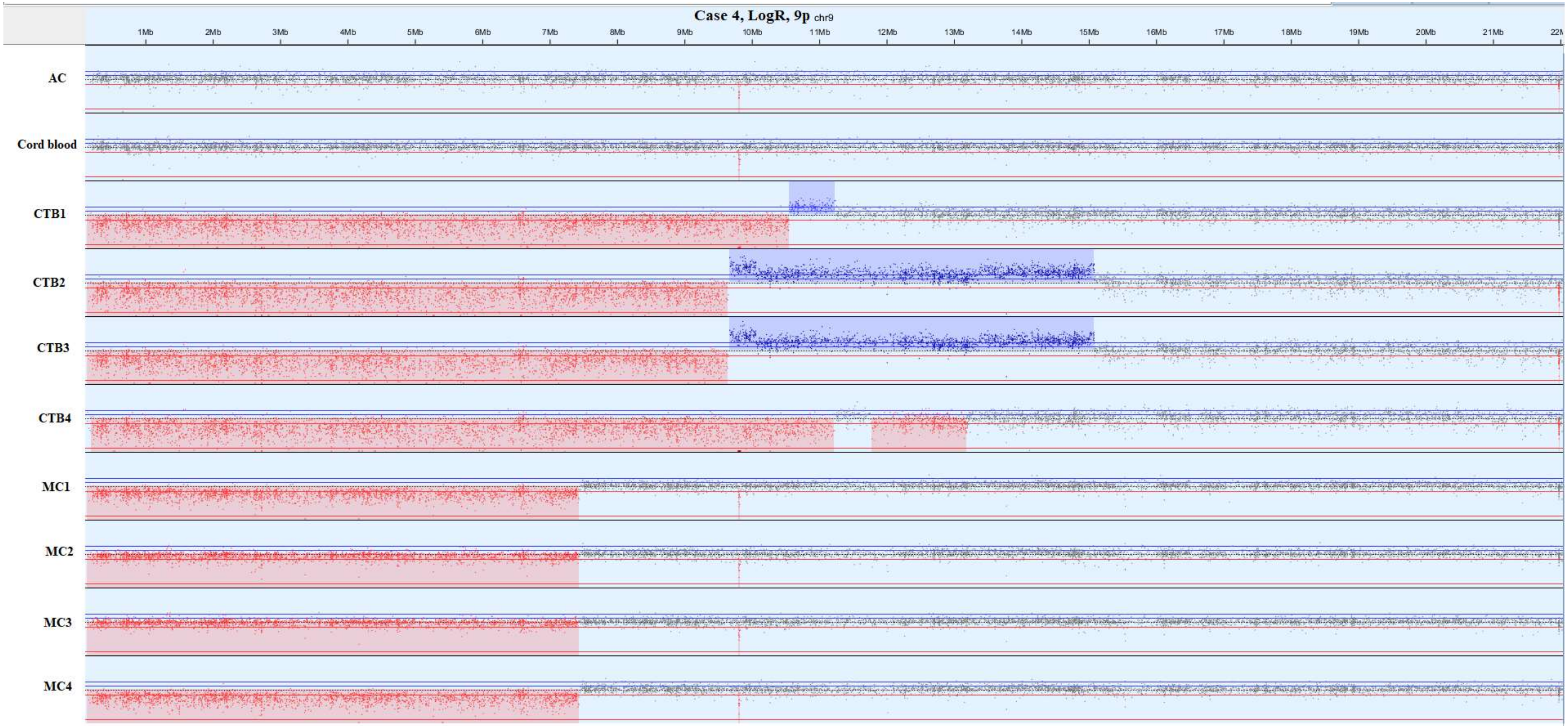
Case 4, LogR, 9p chr9
1Mb
2Mb
3Mb
4Mb
5Mb
6Mb
7Mb
8Mb
9Mb
10Mb
11Mb
12Mb
13Mb
14Mb
15Mb
16Mb
17Mb
18Mb
19Mb
20Mb
21Mb
AC
Cord blood
CTB1
CTB2
CTB3
CTB4
MC1
MC2
MC3
MC4

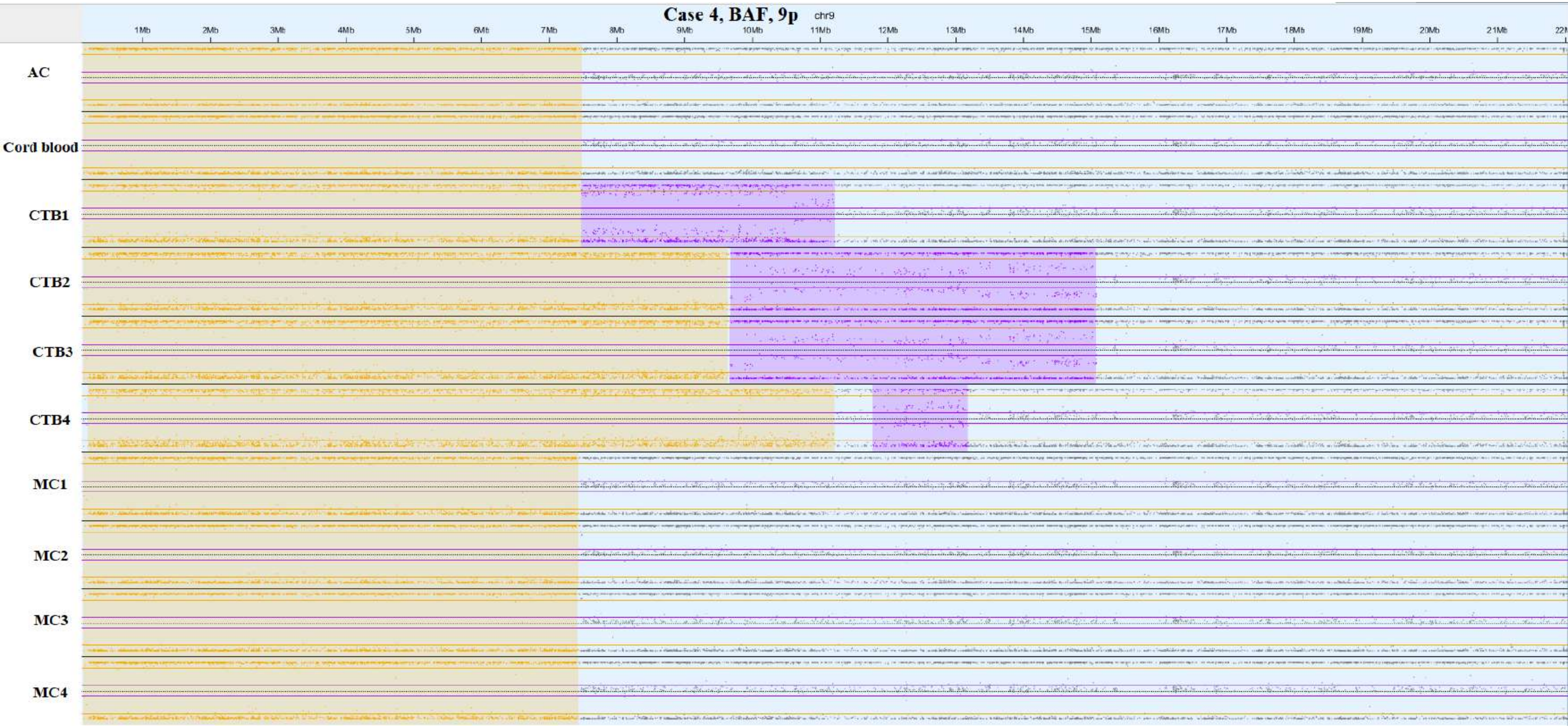

Case 4, BAF, 9p
chr9
1Mb
2Mb
3Mb
4Mb
5Mb
6Mb
7Mb
8Mb
9Mb
10Mb
11Mb
12Mb
13Mb
14Mb
15Mb
16Mb
17Mb
18Mb
19Mb
20Mb
21Mb
AC
Cord blood
CTB1
CTB2
CTB3
CTB4
MC1
MC2
MC3
MC4

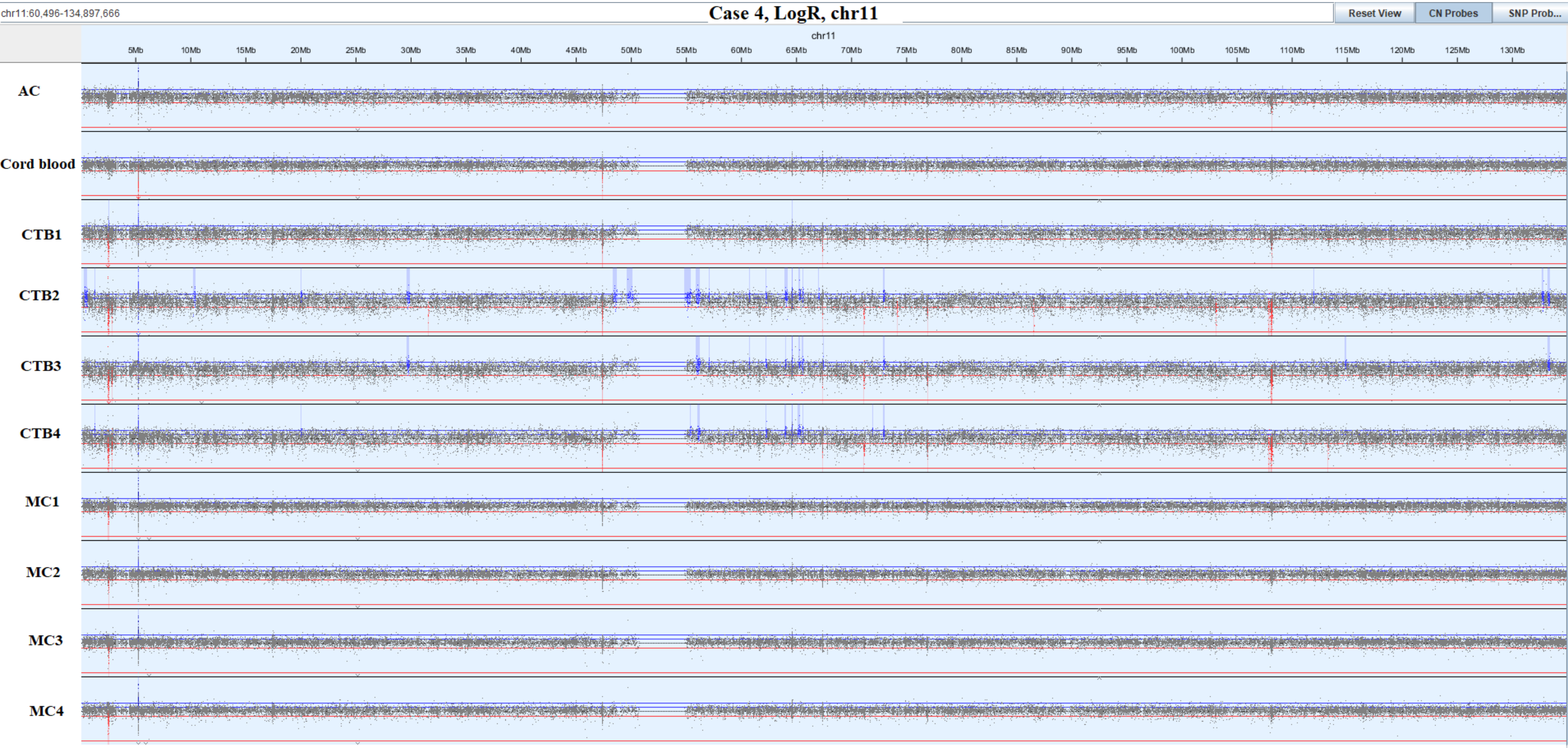

chr11:60,496-134,897,666
Case 4, LogR, chr11
Reset View
CN Probes
SNP Prob...
chr11
5Mb
10Mb
15Mb
20Mb
25Mb
30Mb
35Mb
40Mb
45Mb
50Mb
55Mb
60Mb
65Mb
70Mb
75Mb
80Mb
85Mb
90Mb
95Mb
100Mb
105Mb
110Mb
115Mb
120Mb
125Mb
130Mb
AC
Cord blood
CTB1
CTB2
CTB3
CTB4
MC1
MC2
MC3
MC4

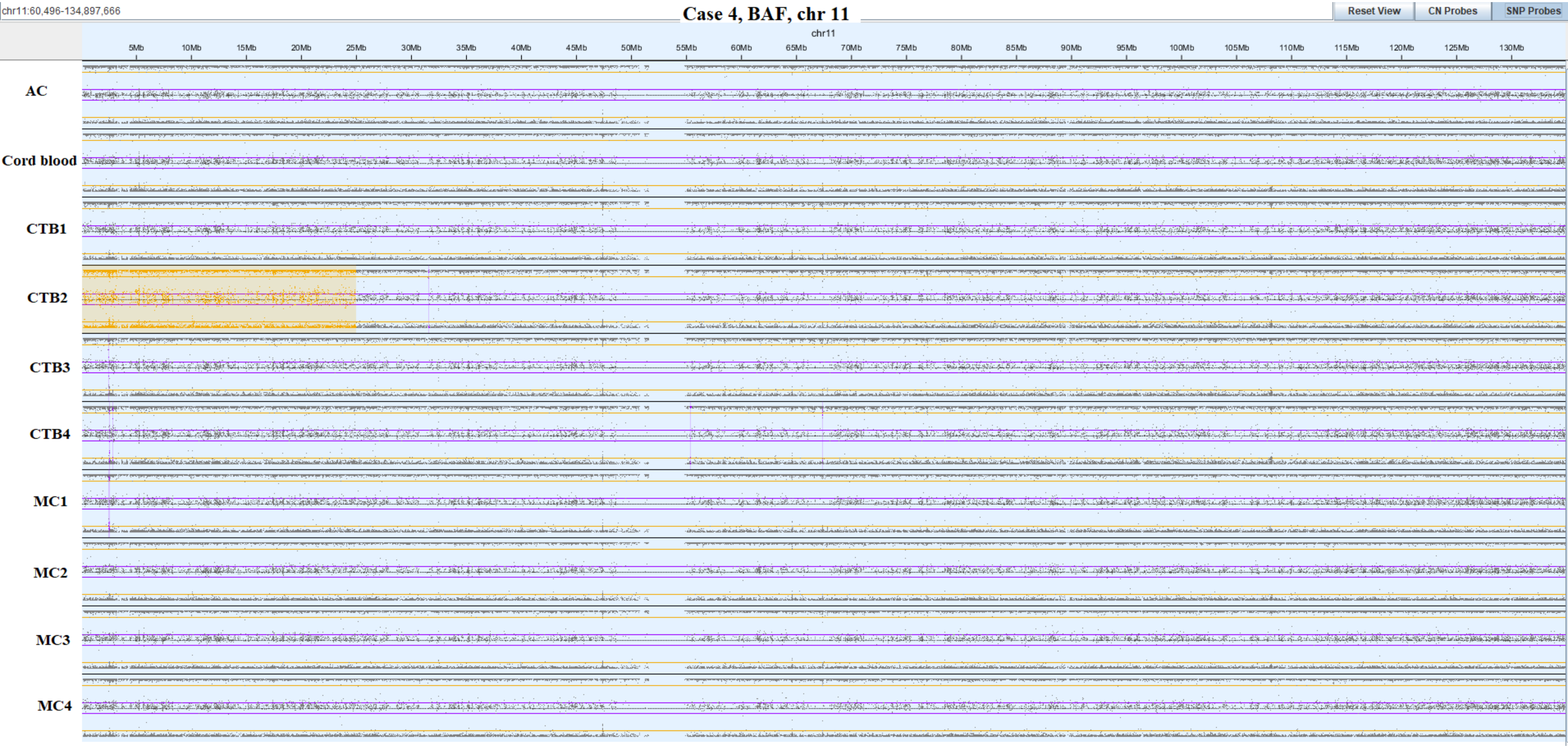
chr11:60,496-134,897,666
Case 4, BAF, chr 11
Reset View
CN Probes
SNP Probes
chr11
5Mb
10Mb
15Mb
20Mb
25Mb
30Mb
35Mb
40Mb
45Mb
50Mb
55Mb
60Mb
65Mb
70Mb
75Mb
80Mb
85Mb
90Mb
95Mb
100Mb
105Mb
110Mb
115Mb
120Mb
125Mb
130Mb
AC
Cord blood
CTB1
CTB2
CTB3
CTB4
MC1
MC2
MC3
MC4

Case 5, NIPT

Fetal fraction: 12%

Mosaic ratio: n.a.

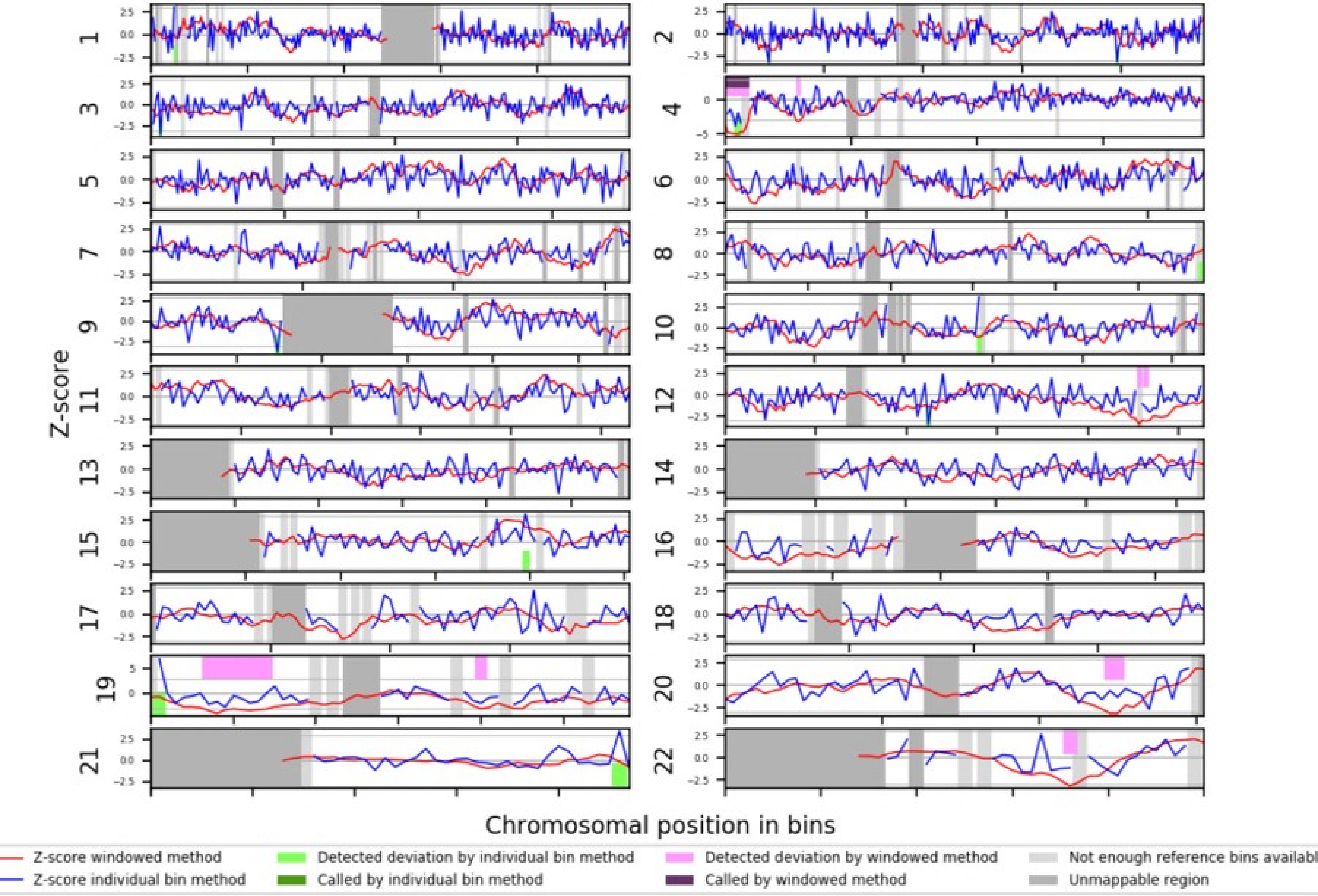

Case 5, LogR and BAF, whole genome

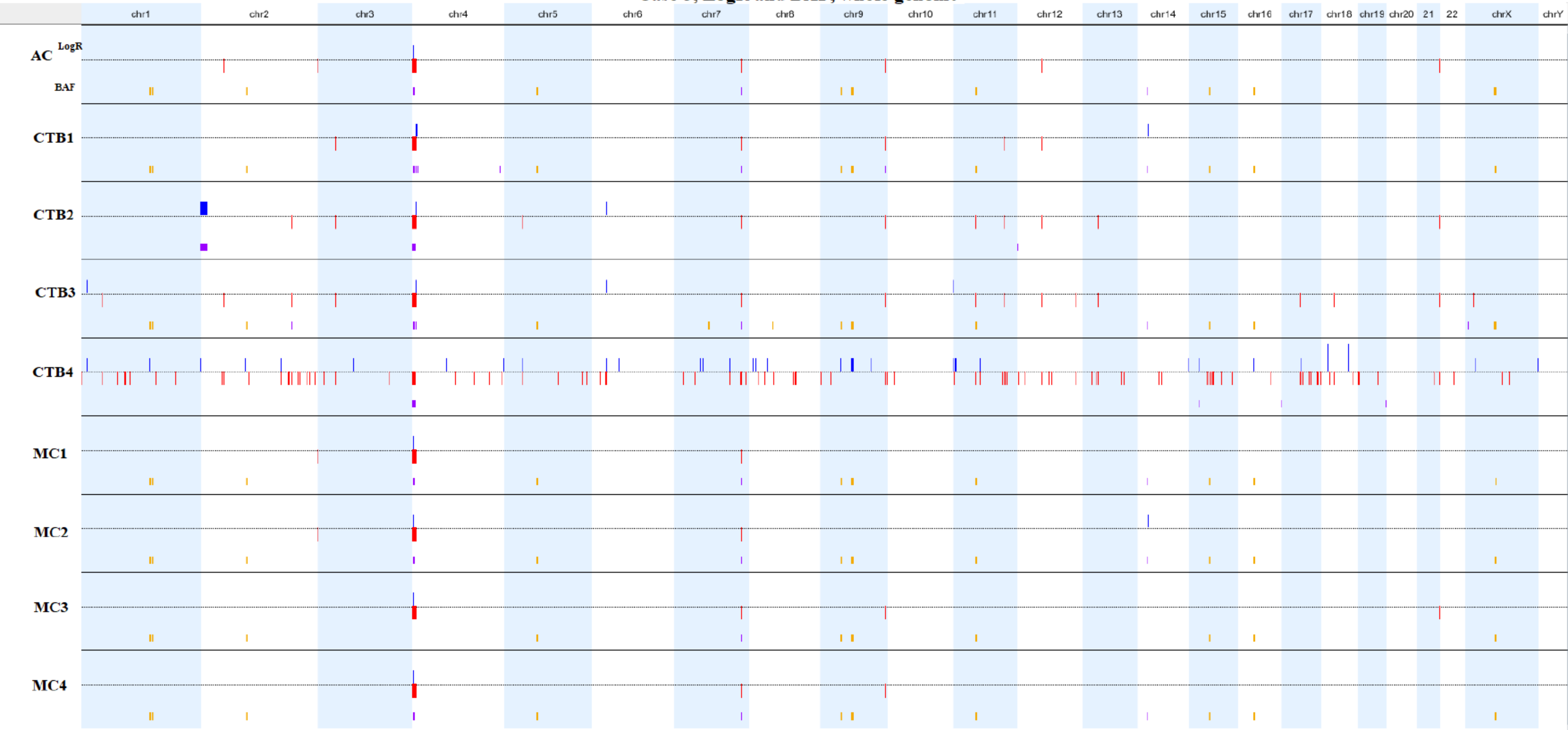

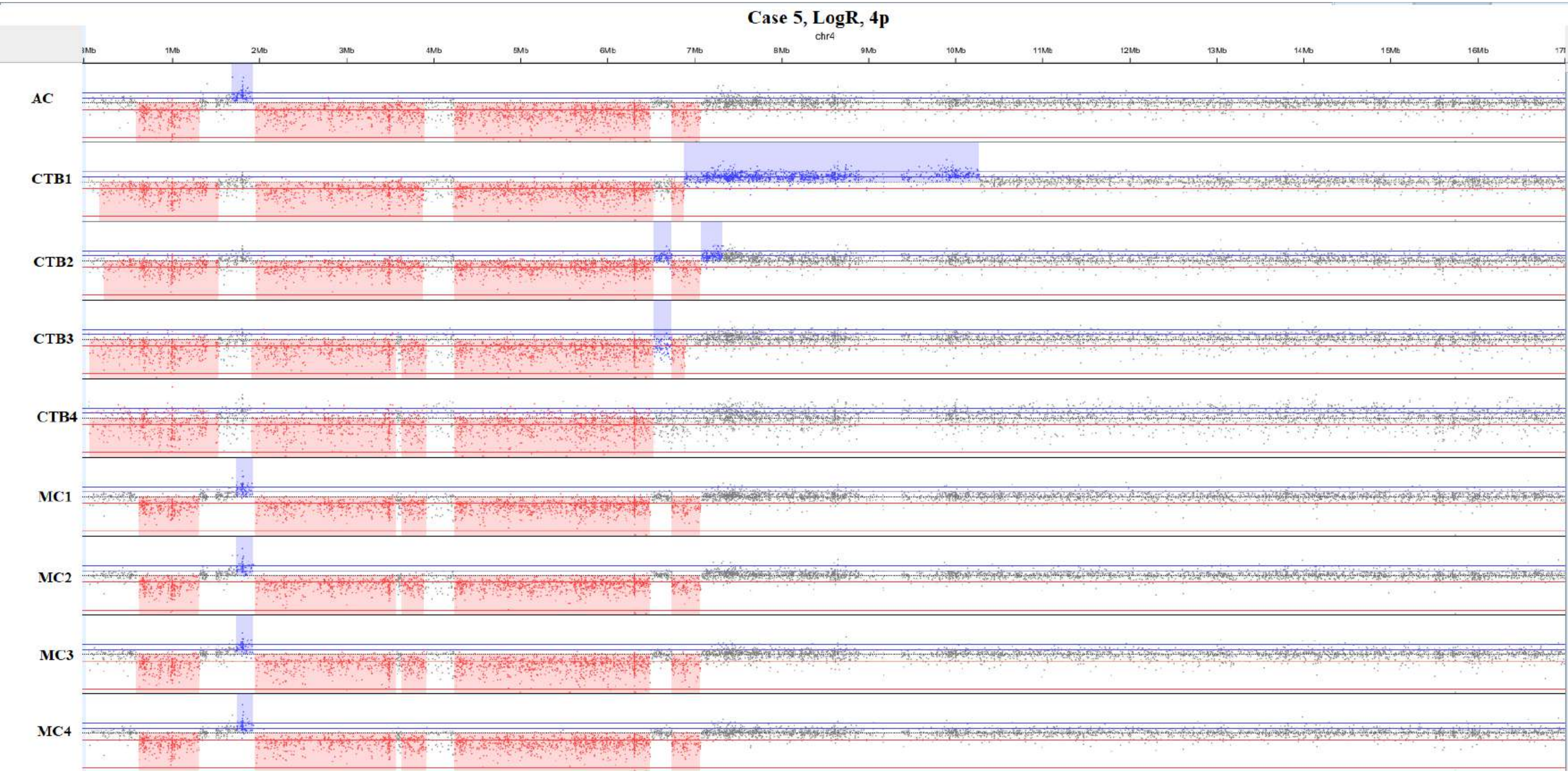
Case 5, LogR, 4p
chr4
0Mb
1Mb
2Mb
3Mb
4Mb
5Mb
6Mb
7Mb
8Mb
9Mb
10Mb
11Mb
12Mb
13Mb
14Mb
15Mb
16Mb
AC
CTB1
CTB2
CTB3
CTB4
MC1
MC2
MC3
MC4

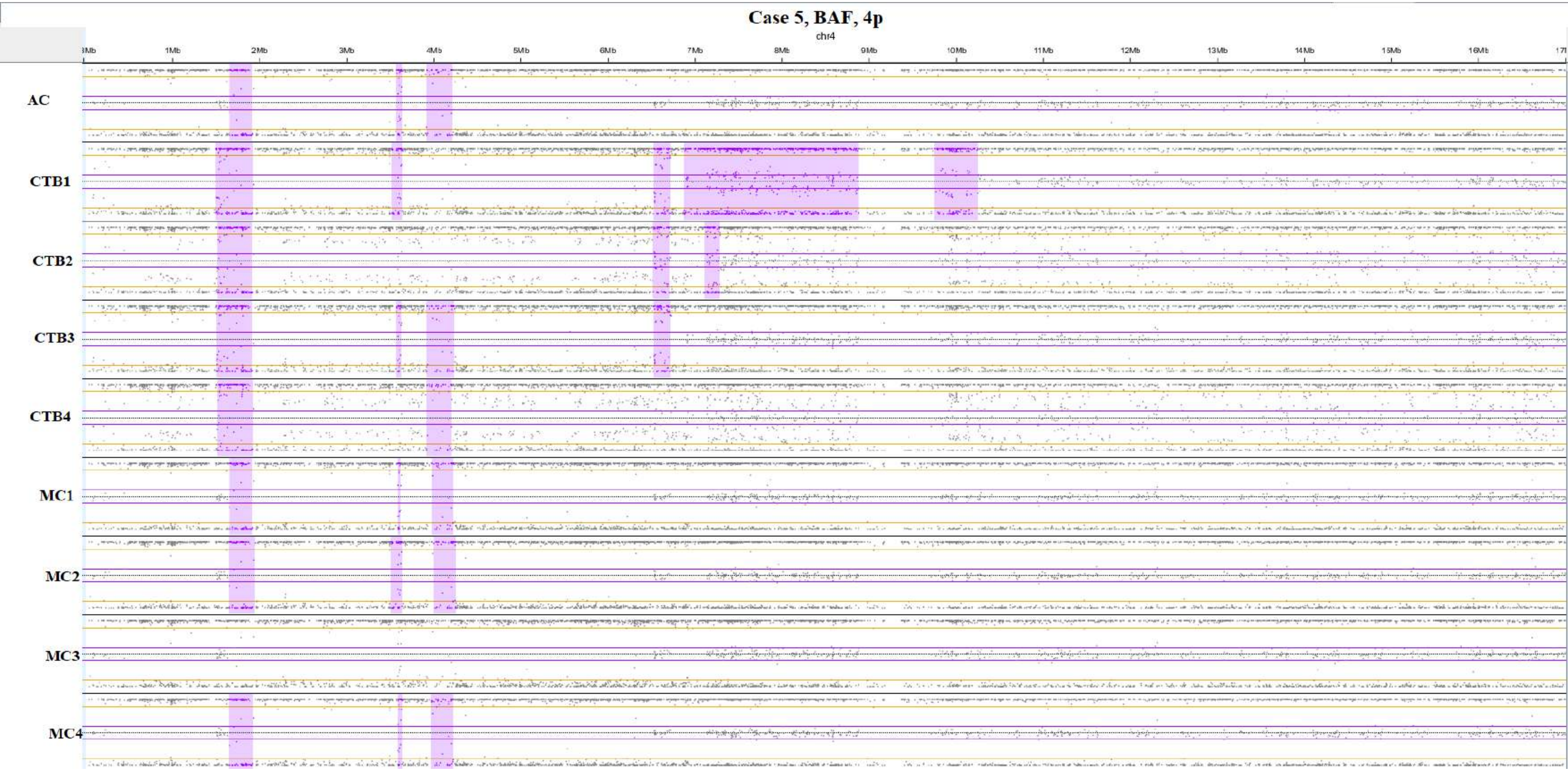
Case 5, BAF, 4p
chr4
0Mb
1Mb
2Mb
3Mb
4Mb
5Mb
6Mb
7Mb
8Mb
9Mb
10Mb
11Mb
12Mb
13Mb
14Mb
15Mb
16Mb
AC
CTB1
CTB2
CTB3
CTB4
MC1
MC2
MC3
MC4

Case 6, NIPT

Fetal fraction: 5%

Mosaic ratio: n.a.

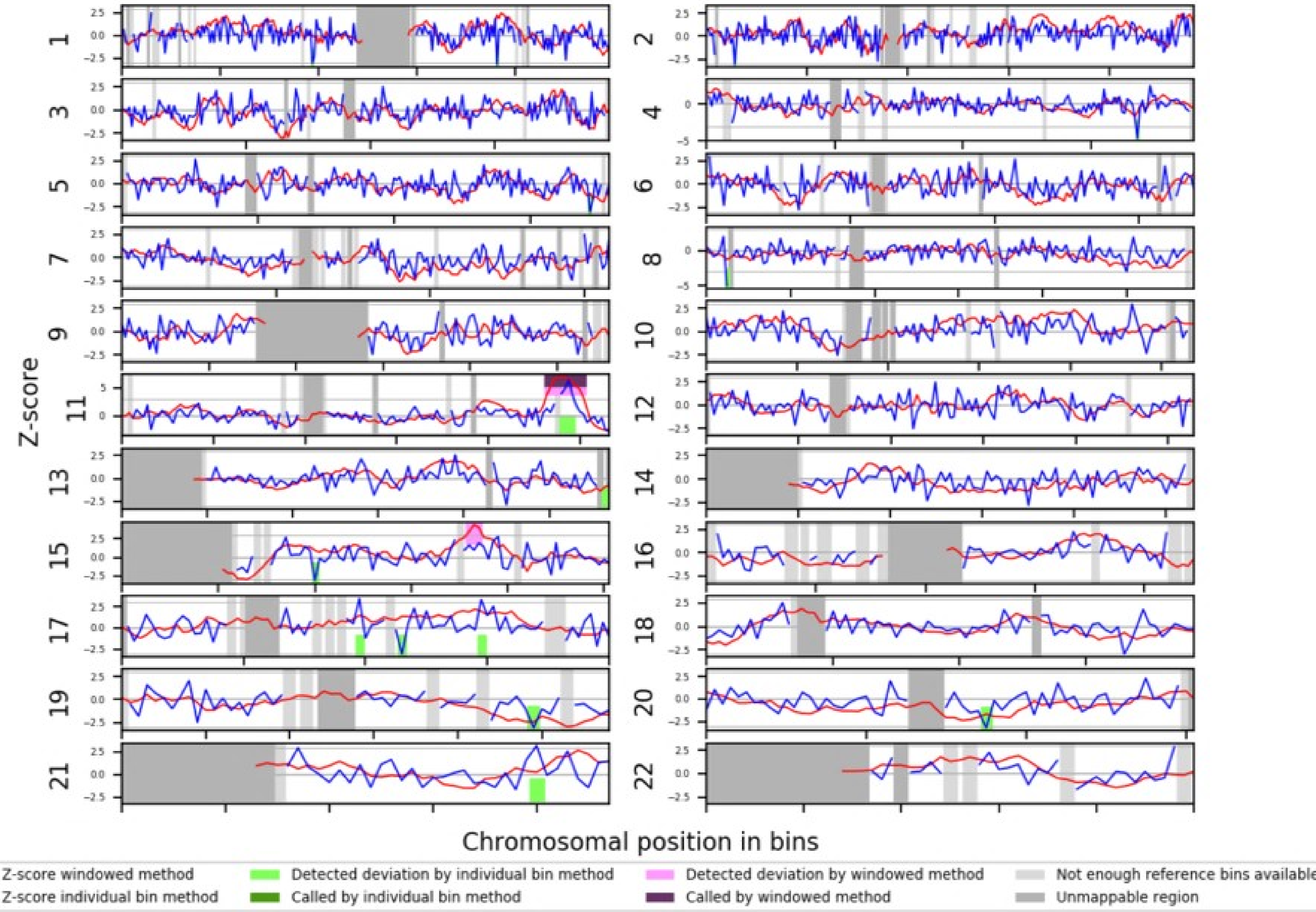

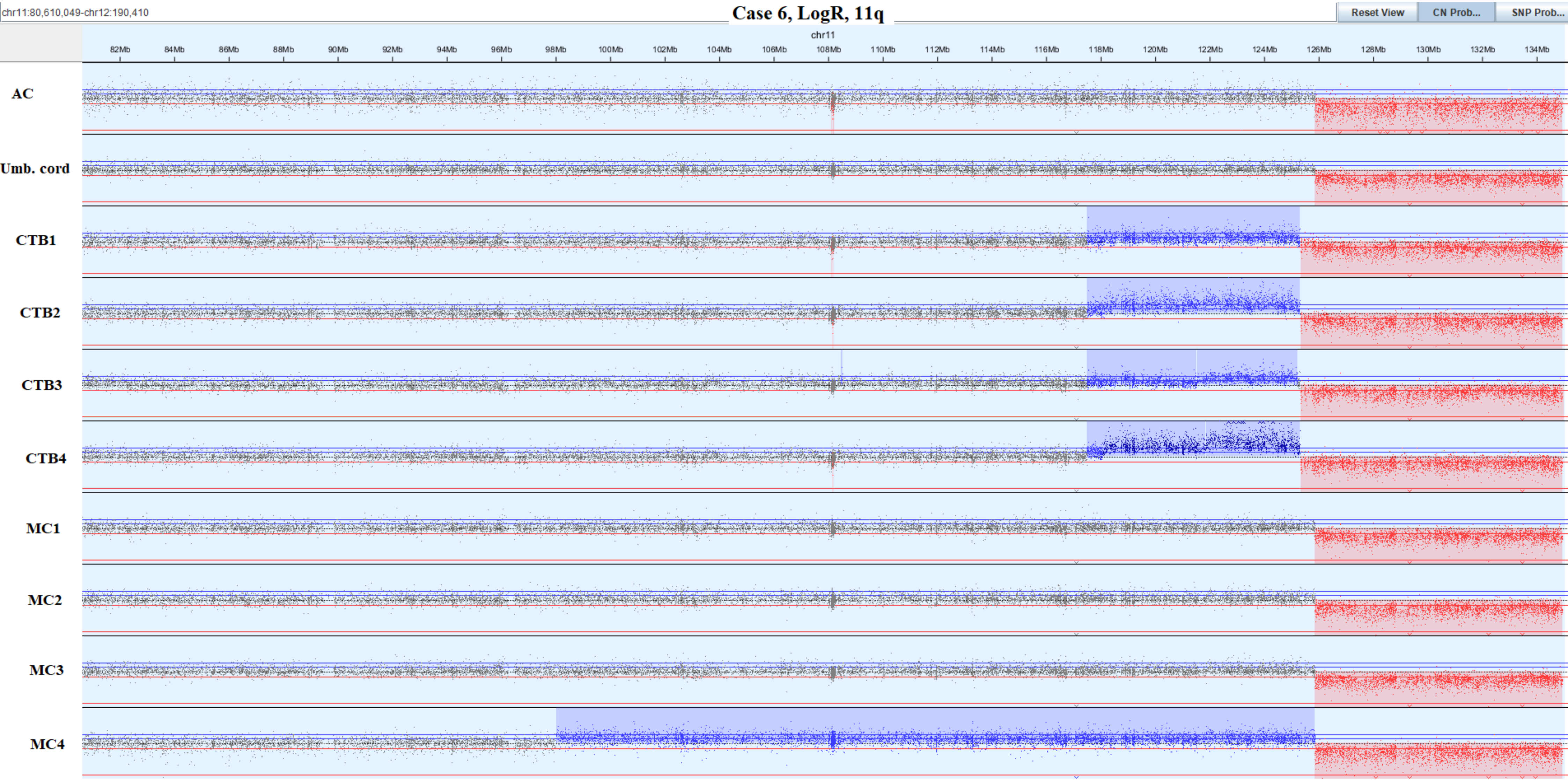

chr11:80,610,049-chr12:190,410
Case 6, LogR, 11q
Reset View
CN Prob...
SNP Prob...
chr11
82Mb
84Mb
86Mb
88Mb
90Mb
92Mb
94Mb
96Mb
98Mb
100Mb
102Mb
104Mb
106Mb
108Mb
110Mb
112Mb
114Mb
116Mb
118Mb
120Mb
122Mb
124Mb
126Mb
128Mb
130Mb
132Mb
134Mb
AC
Umb. cord
CTB1
CTB2
CTB3
CTB4
MC1
MC2
MC3
MC4

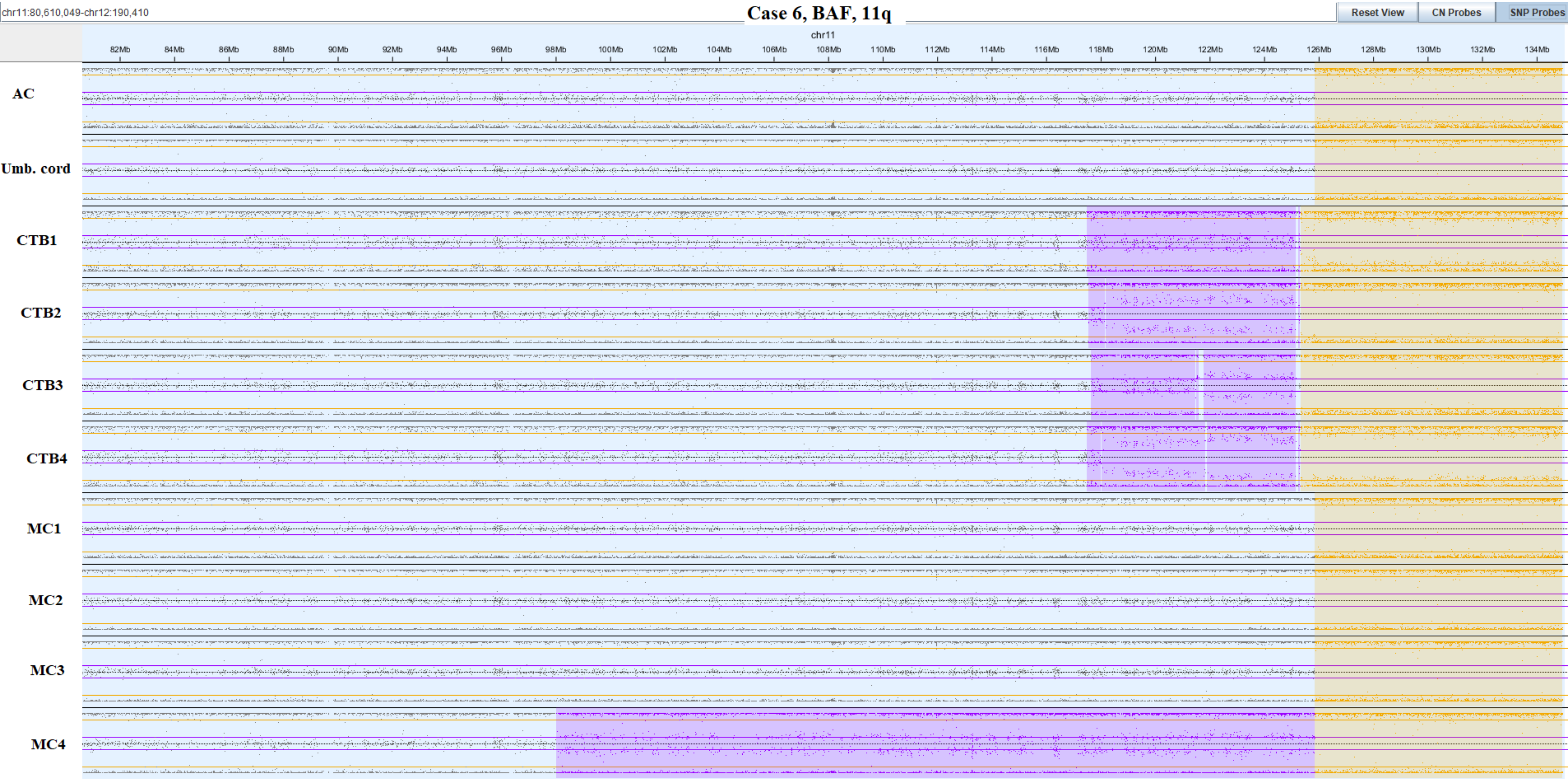

chr11:80,610,049-chr12:190,410
Case 6, BAF, 11q
Reset View
CN Probes
SNP Probes
chr11
82Mb
84Mb
86Mb
88Mb
90Mb
92Mb
94Mb
96Mb
98Mb
100Mb
102Mb
104Mb
106Mb
108Mb
110Mb
112Mb
114Mb
116Mb
118Mb
120Mb
122Mb
124Mb
126Mb
128Mb
130Mb
132Mb
134Mb
AC
Umb. cord
CTB1
CTB2
CTB3
CTB4
MC1
MC2
MC3
MC4

Case 7, NIPT

Fetal fraction: 9%

Mosaic ratio: 0.44 (9p gain)

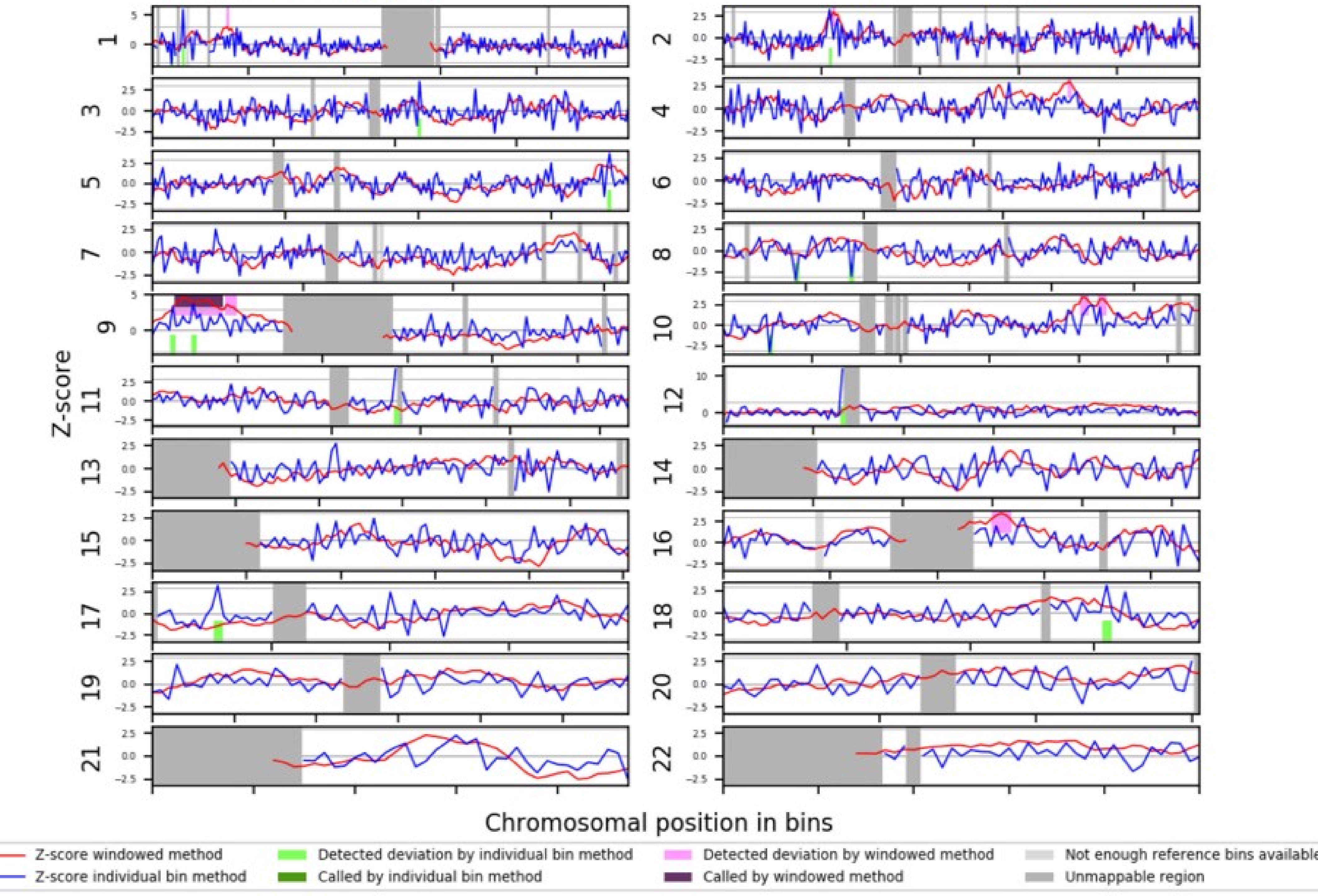

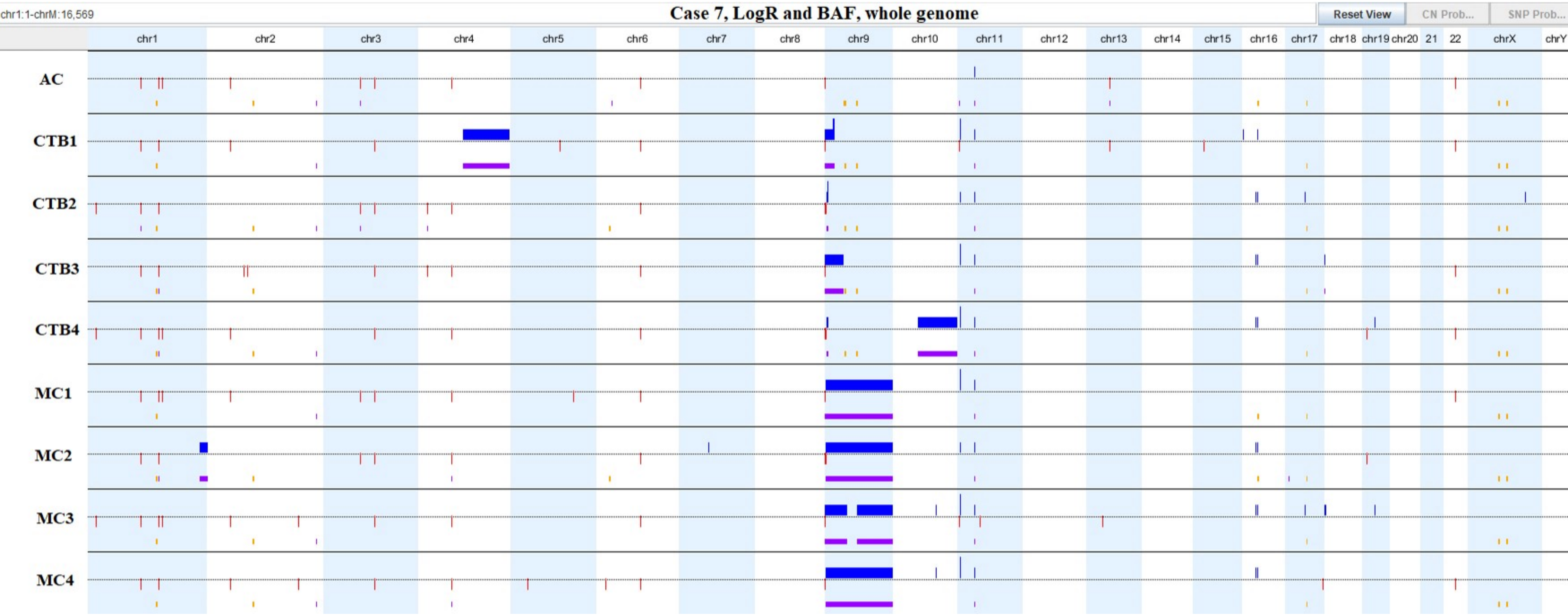
chr1:1-chrM:16,569
Case 7, LogR and BAF, whole genome
Reset View
CN Prob...
SNP Prob...
chr1
chr2
chr3
chr4
chr5
chr6
chr7
chr8
chr9
chr10
chr11
chr12
chr13
chr14
chr15
chr16
chr17
chr18
chr19
chr20
21
22
chrX
chrY
AC
CTB1
CTB2
CTB3
CTB4
MC1
MC2
MC3
MC4

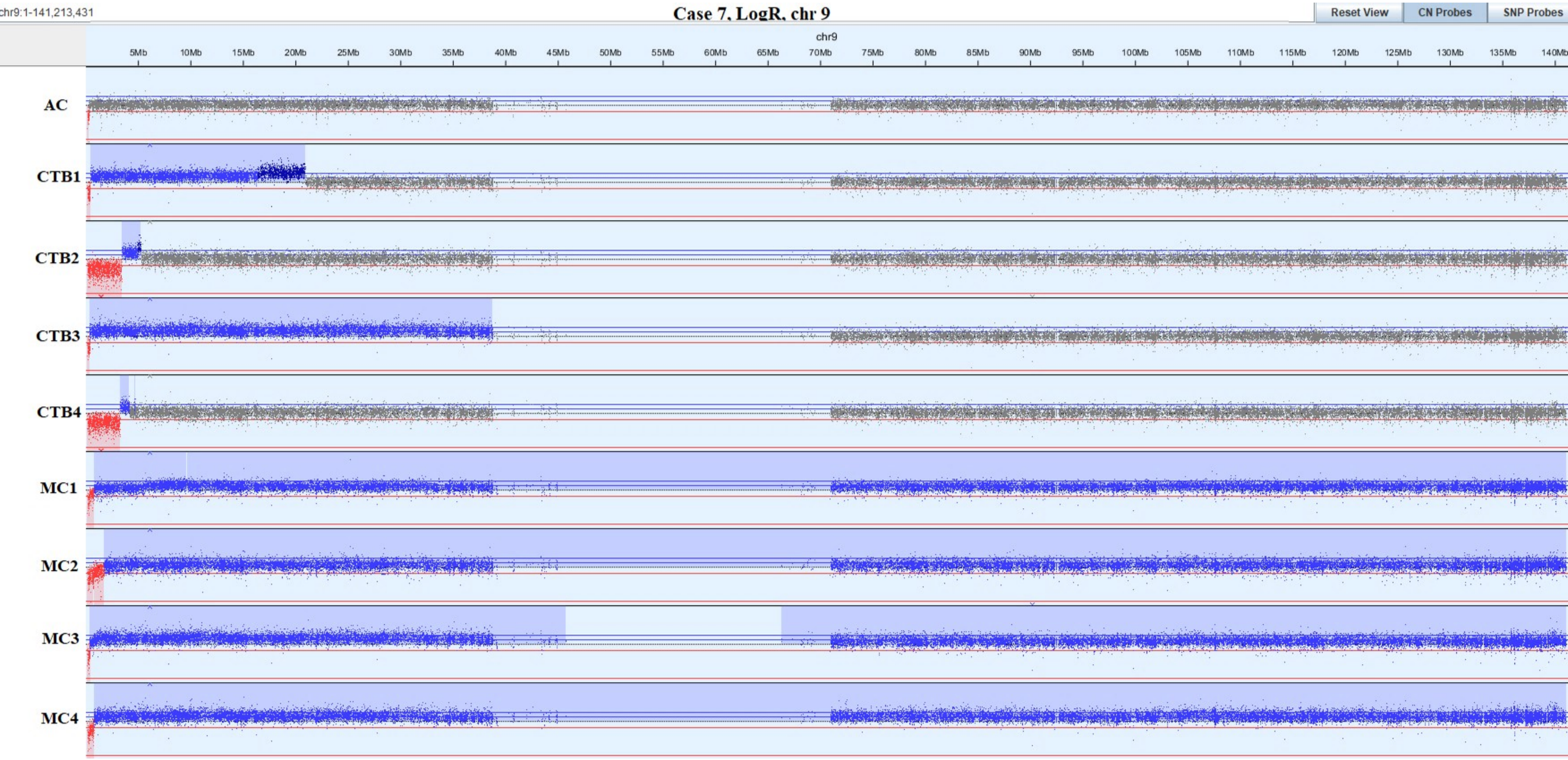

chr9:1-141,213,431
Case 7, LogR, chr 9
Reset View
CN Probes
SNP Probes
chr9
5Mb 10Mb 15Mb 20Mb 25Mb 30Mb 35Mb 40Mb 45Mb 50Mb 55Mb 60Mb 65Mb 70Mb 75Mb 80Mb 85Mb 90Mb 95Mb 100Mb 105Mb 110Mb 115Mb 120Mb 125Mb 130Mb 135Mb 140Mb
AC
CTB1
CTB2
CTB3
CTB4
MC1
MC2
MC3
MC4

Case 7, BAF, chr 9

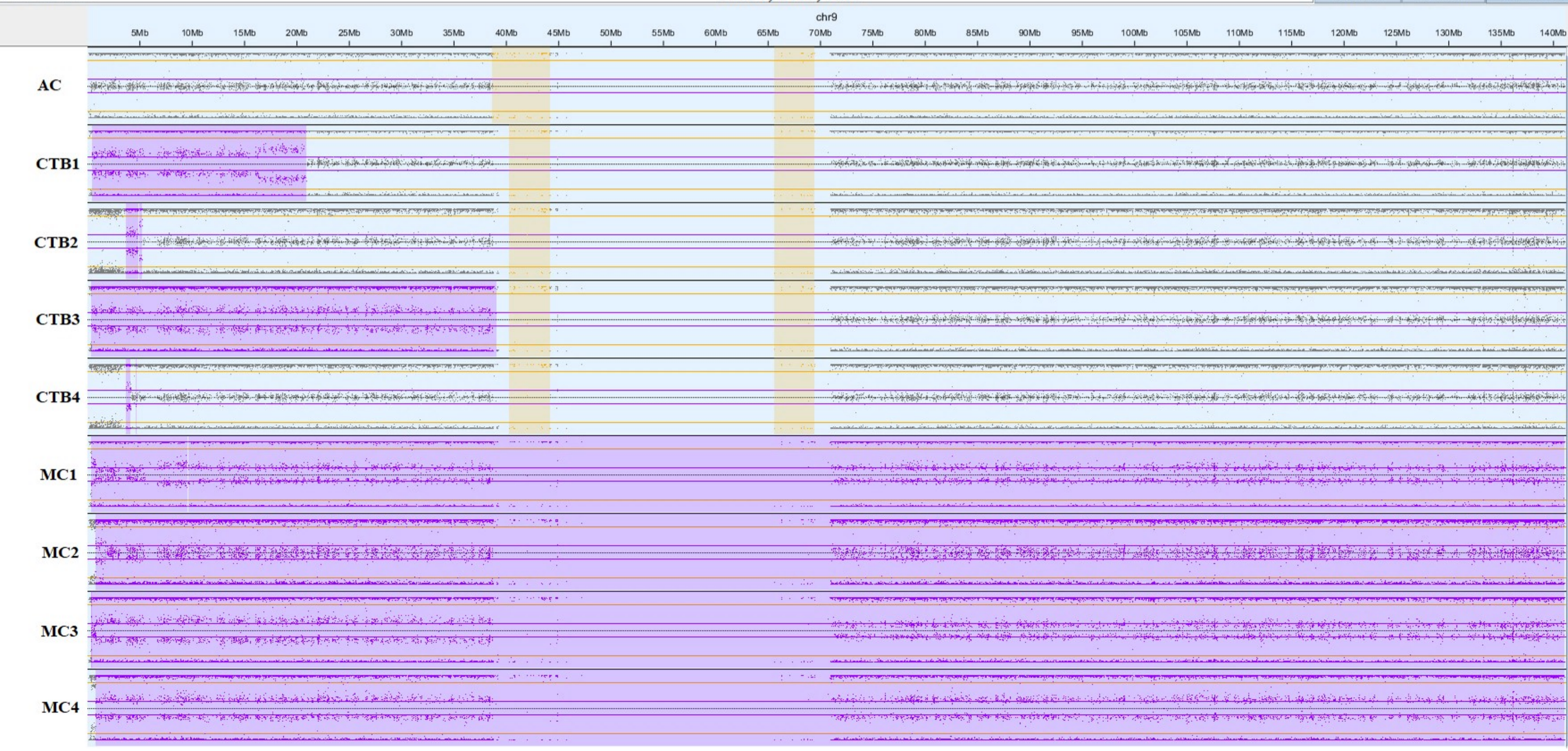

Case 8, NIPT

Fetal fraction: 11%

Mosaic ratio: 1.04 (4p gain) and 1.25 (11q loss)

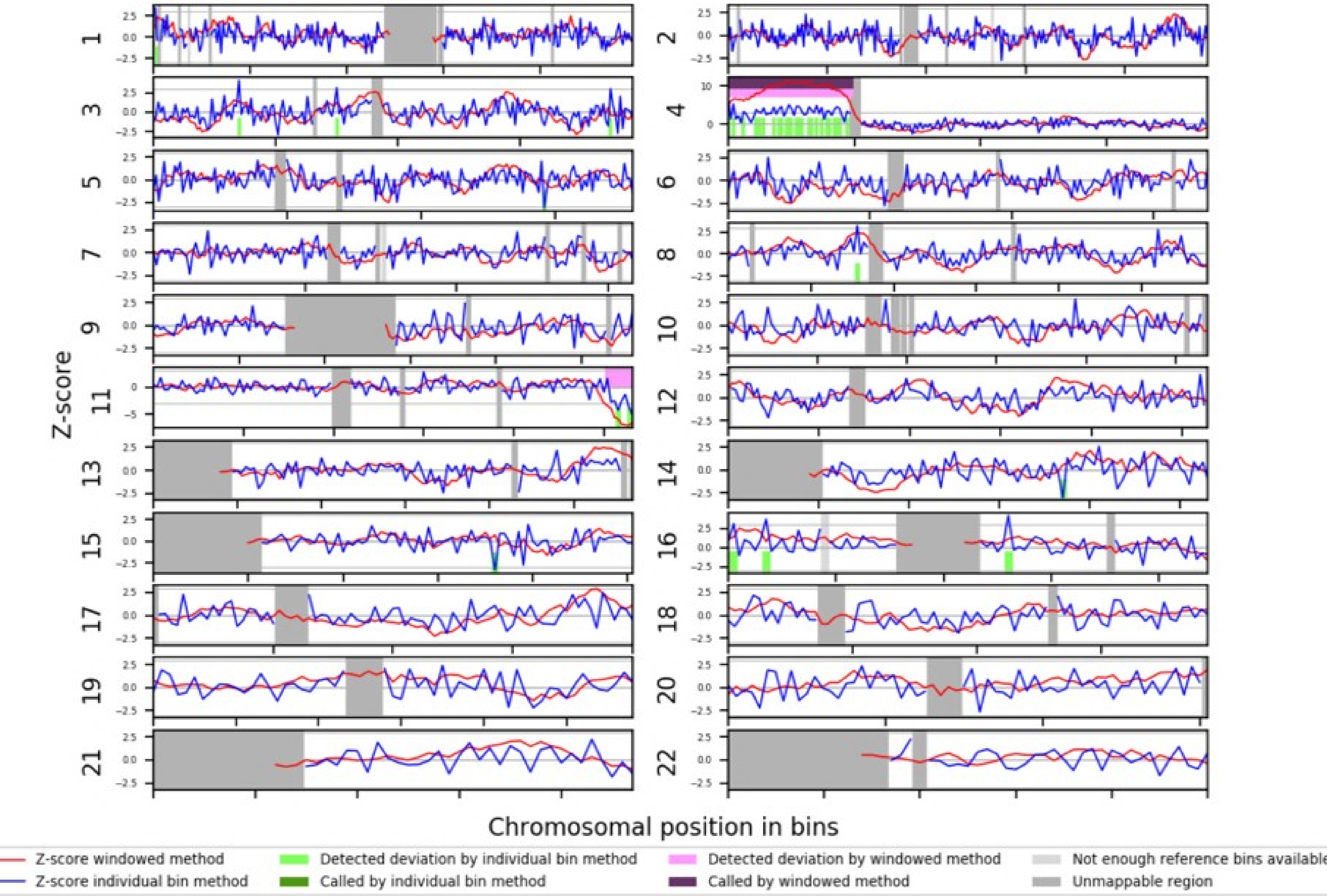

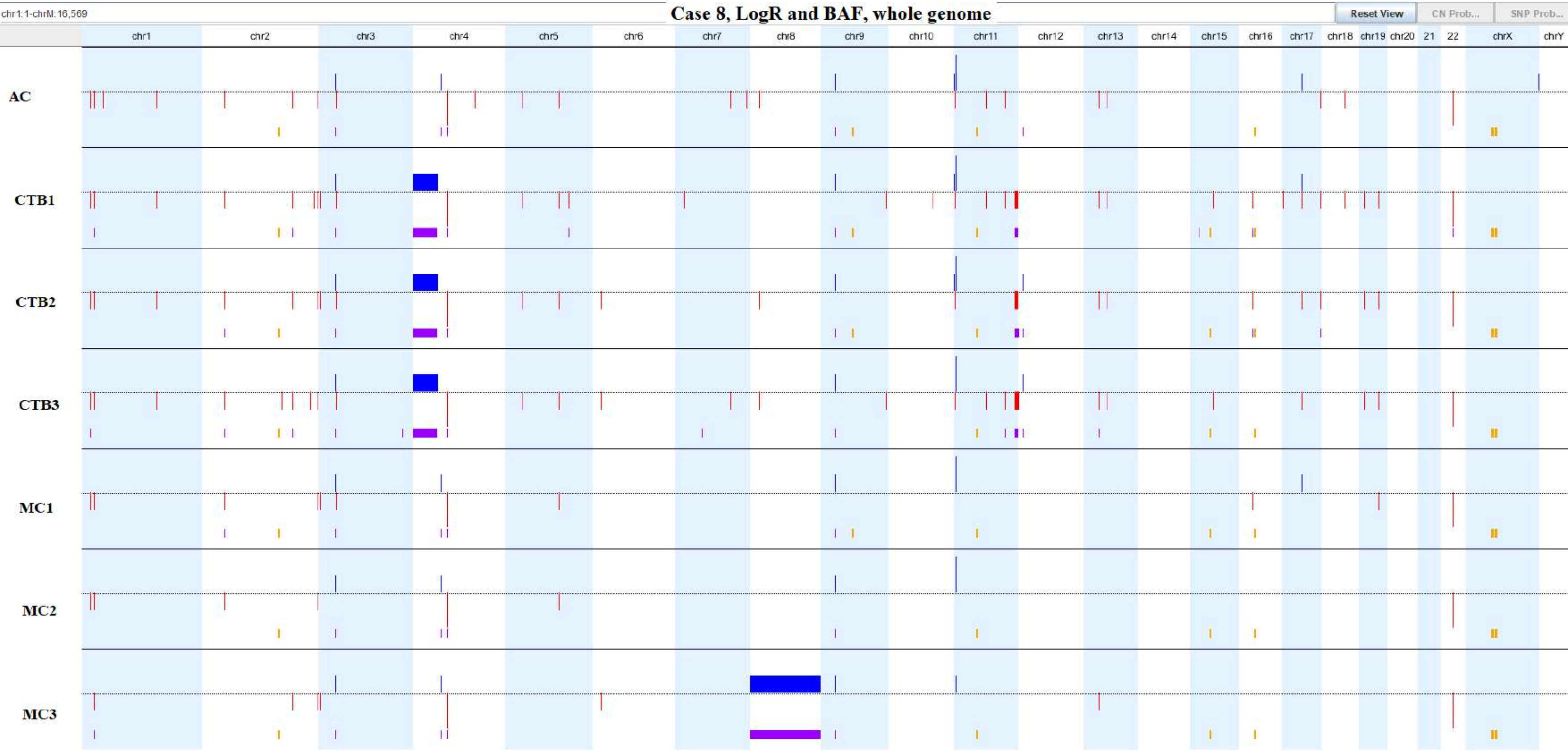

chr1:1-chrM:16,569
Case 8, LogR and BAF, whole genome
Reset View
CN Prob...
SNP Prob...
chr1
chr2
chr3
chr4
chr5
chr6
chr7
chr8
chr9
chr10
chr11
chr12
chr13
chr14
chr15
chr16
chr17
chr18
chr19
chr20
21
22
chrX
chrY
AC
CTB1
CTB2
CTB3
MC1
MC2
MC3

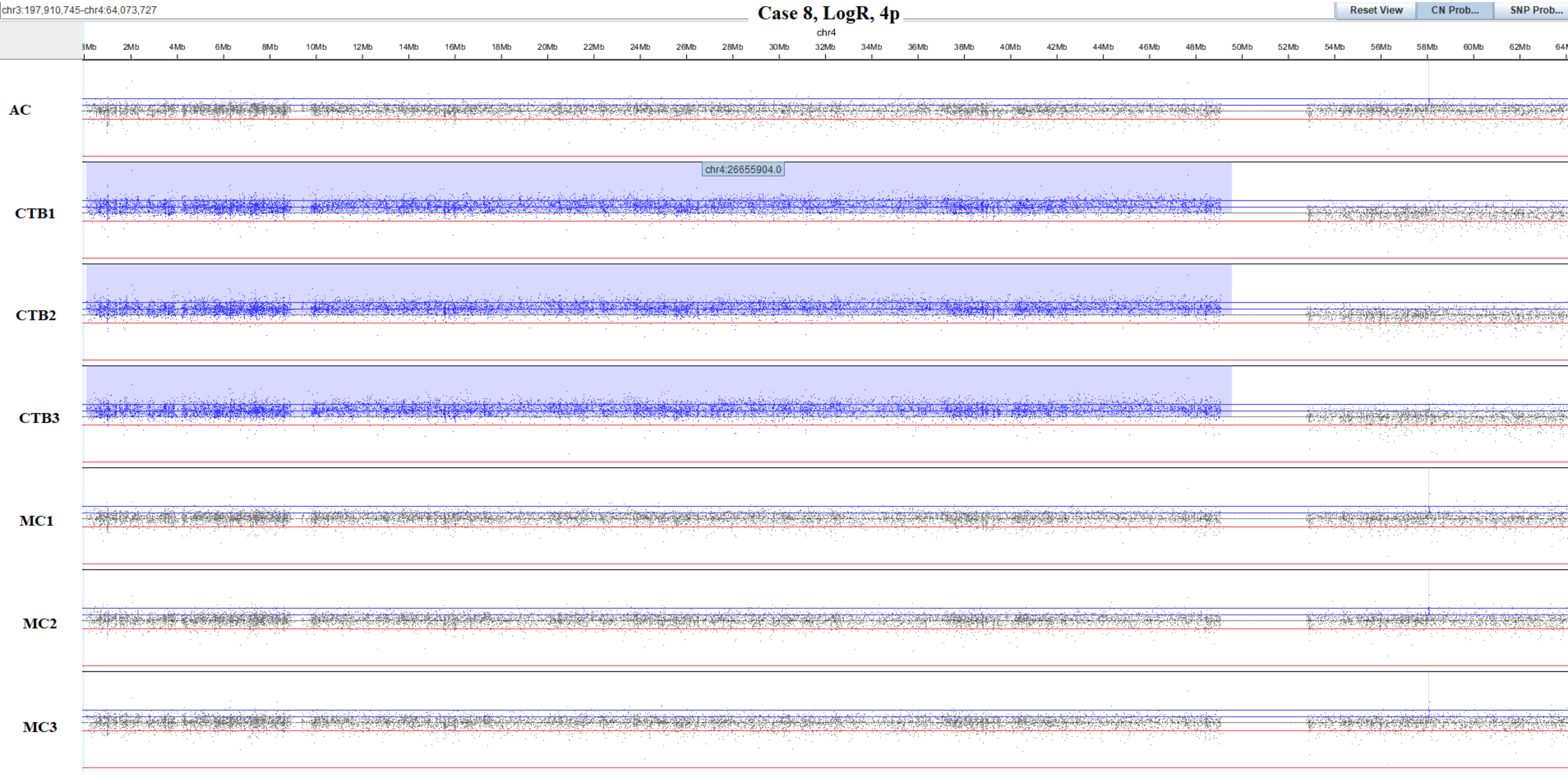
chr3:197,910,745-chr4:64,073,727
Case 8, LogR, 4p
Reset View
CN Prob...
SNP Prob...
chr4
2Mb
4Mb
6Mb
8Mb
10Mb
12Mb
14Mb
16Mb
18Mb
20Mb
22Mb
24Mb
26Mb
28Mb
30Mb
32Mb
34Mb
36Mb
38Mb
40Mb
42Mb
44Mb
46Mb
48Mb
50Mb
52Mb
54Mb
56Mb
58Mb
60Mb
62Mb
AC
CTB1
chr4:26655904.0
CTB2
CTB3
MC1
MC2
MC3

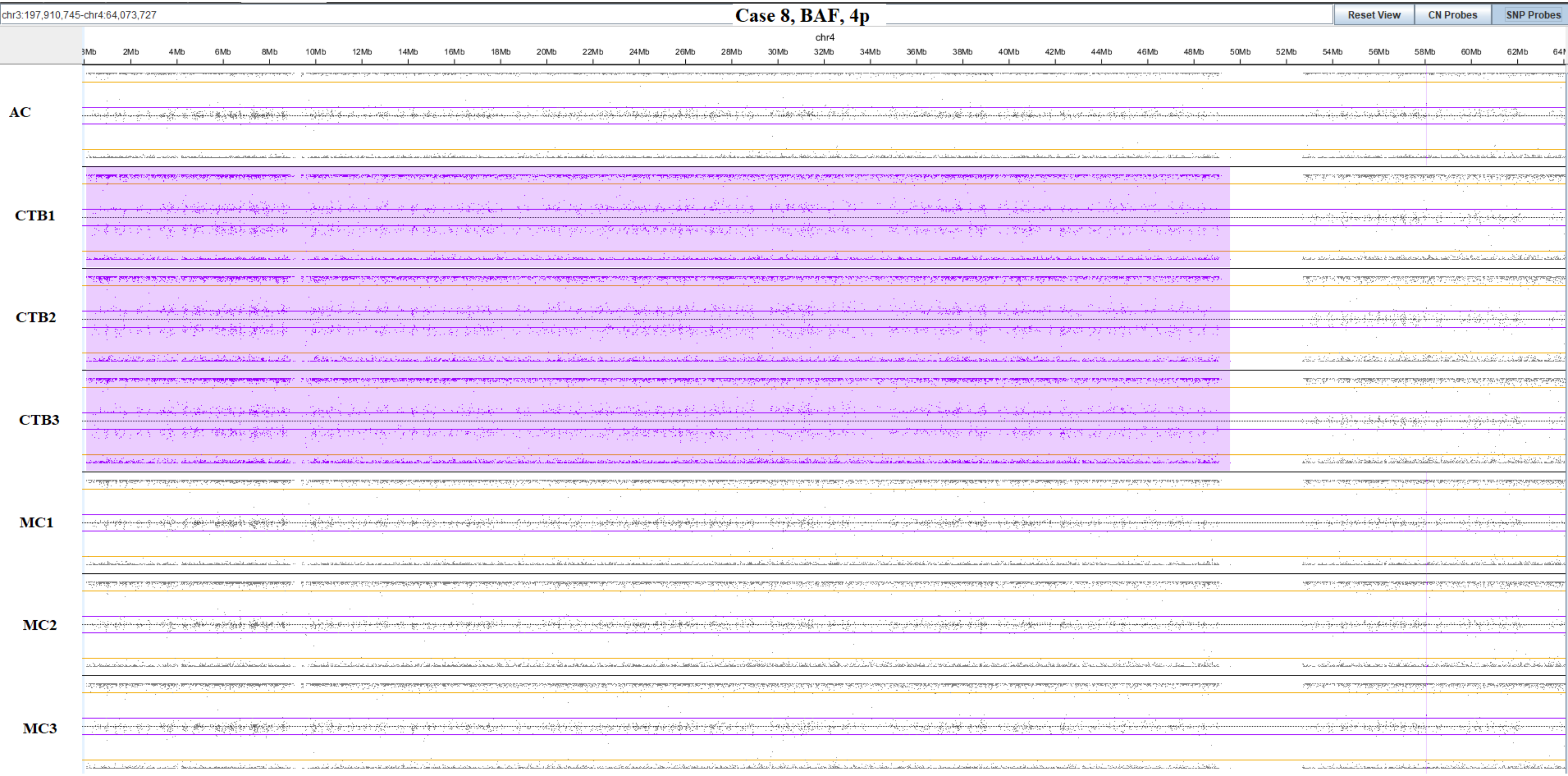

chr3:197,910,745-chr4:64,073,727
Case 8, BAF, 4p
Reset View
CN Probes
SNP Probes
chr4
2Mb
4Mb
6Mb
8Mb
10Mb
12Mb
14Mb
16Mb
18Mb
20Mb
22Mb
24Mb
26Mb
28Mb
30Mb
32Mb
34Mb
36Mb
38Mb
40Mb
42Mb
44Mb
46Mb
48Mb
50Mb
52Mb
54Mb
56Mb
58Mb
60Mb
62Mb
AC
CTB1
CTB2
CTB3
MC1
MC2
MC3

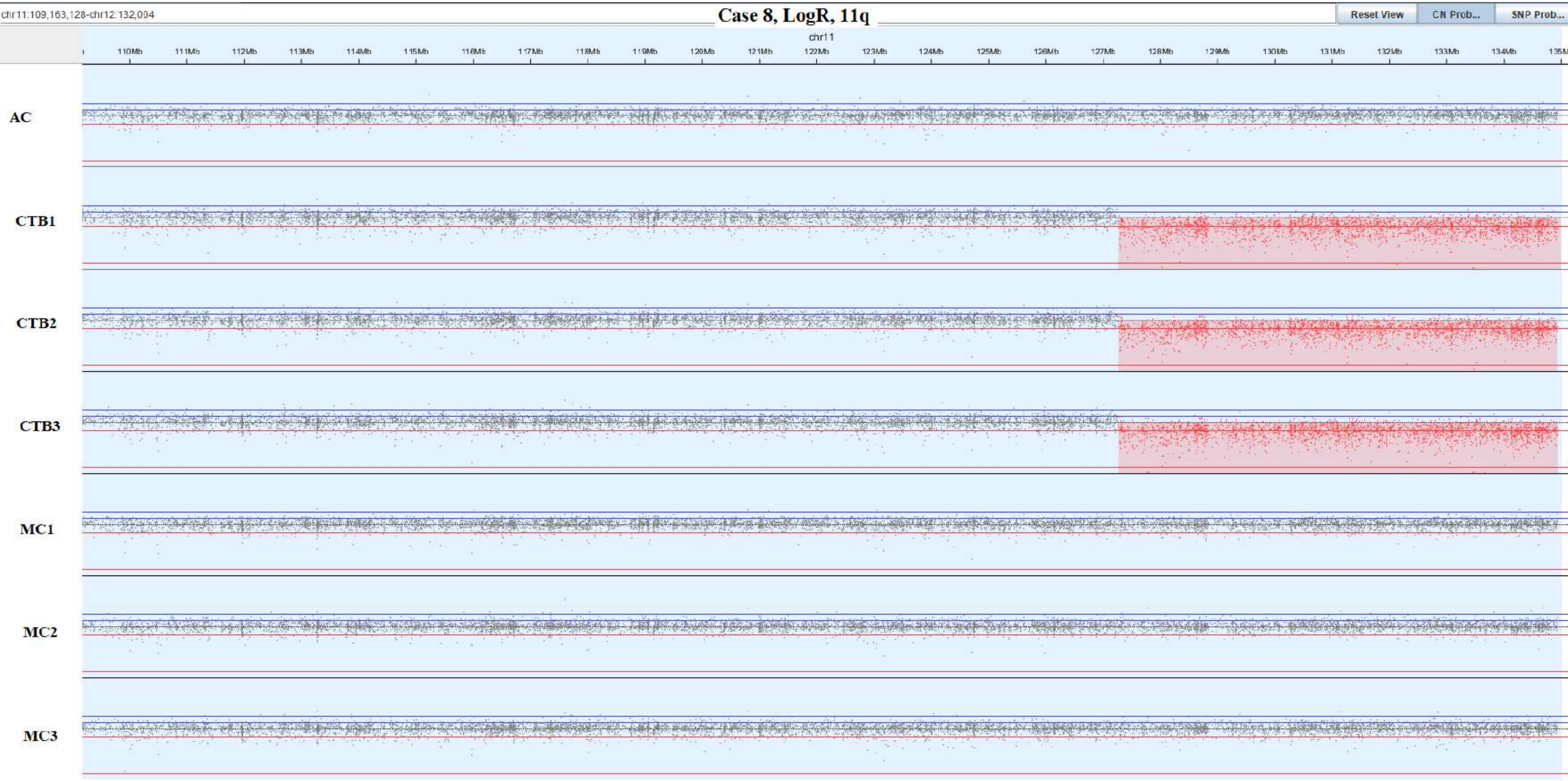
chr11:109,163,128-chr12:132,004
Case 8, LogR, 11q
Reset View
CN Prob...
SNP Prob...
chr11
110Mb
111Mb
112Mb
113Mb
114Mb
115Mb
116Mb
117Mb
118Mb
119Mb
120Mb
121Mb
122Mb
123Mb
124Mb
125Mb
126Mb
127Mb
128Mb
129Mb
130Mb
131Mb
132Mb
133Mb
134Mb
AC
CTB1
CTB2
CTB3
MC1
MC2
MC3

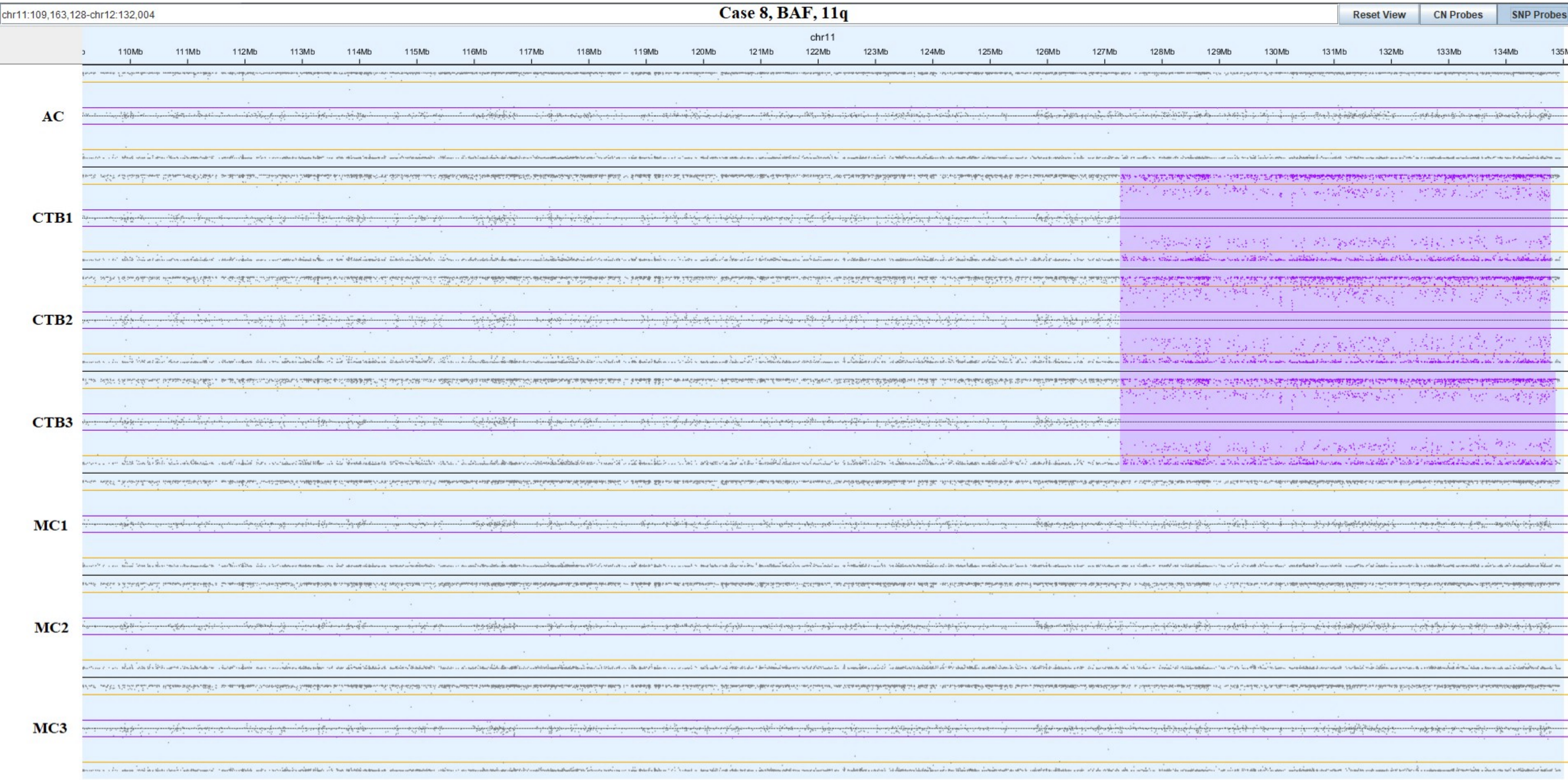

chr11:109,163,128-chr12:132,004
Case 8, BAF, 11q
Reset View
CN Probes
SNP Probes
chr11
110Mb
111Mb
112Mb
113Mb
114Mb
115Mb
116Mb
117Mb
118Mb
119Mb
120Mb
121Mb
122Mb
123Mb
124Mb
125Mb
126Mb
127Mb
128Mb
129Mb
130Mb
131Mb
132Mb
133Mb
134Mb
AC
CTB1
CTB2
CTB3
MC1
MC2
MC3

Case 9, NIPT

Fetal fraction: 7%

Mosaic ratio: n.a.

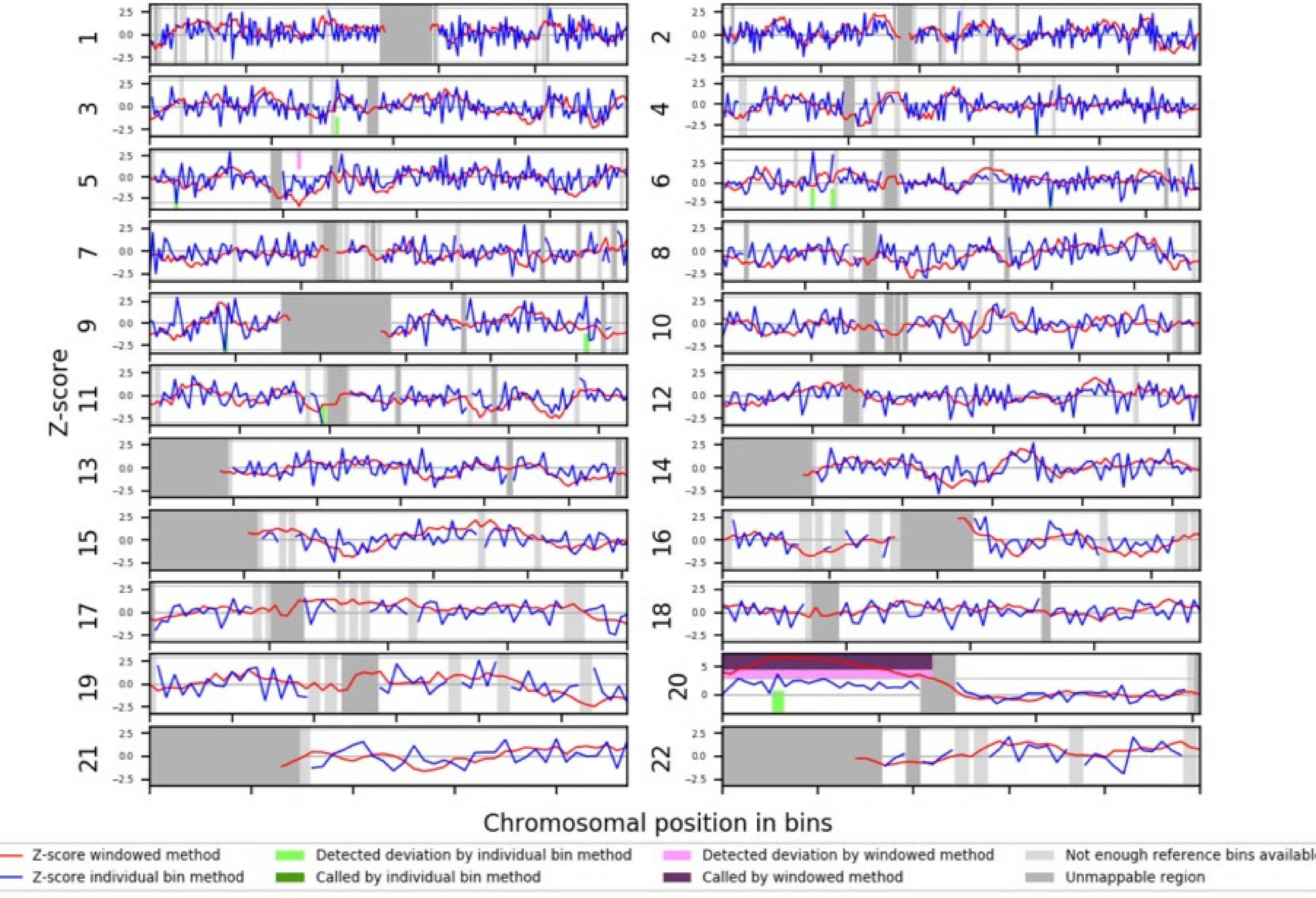

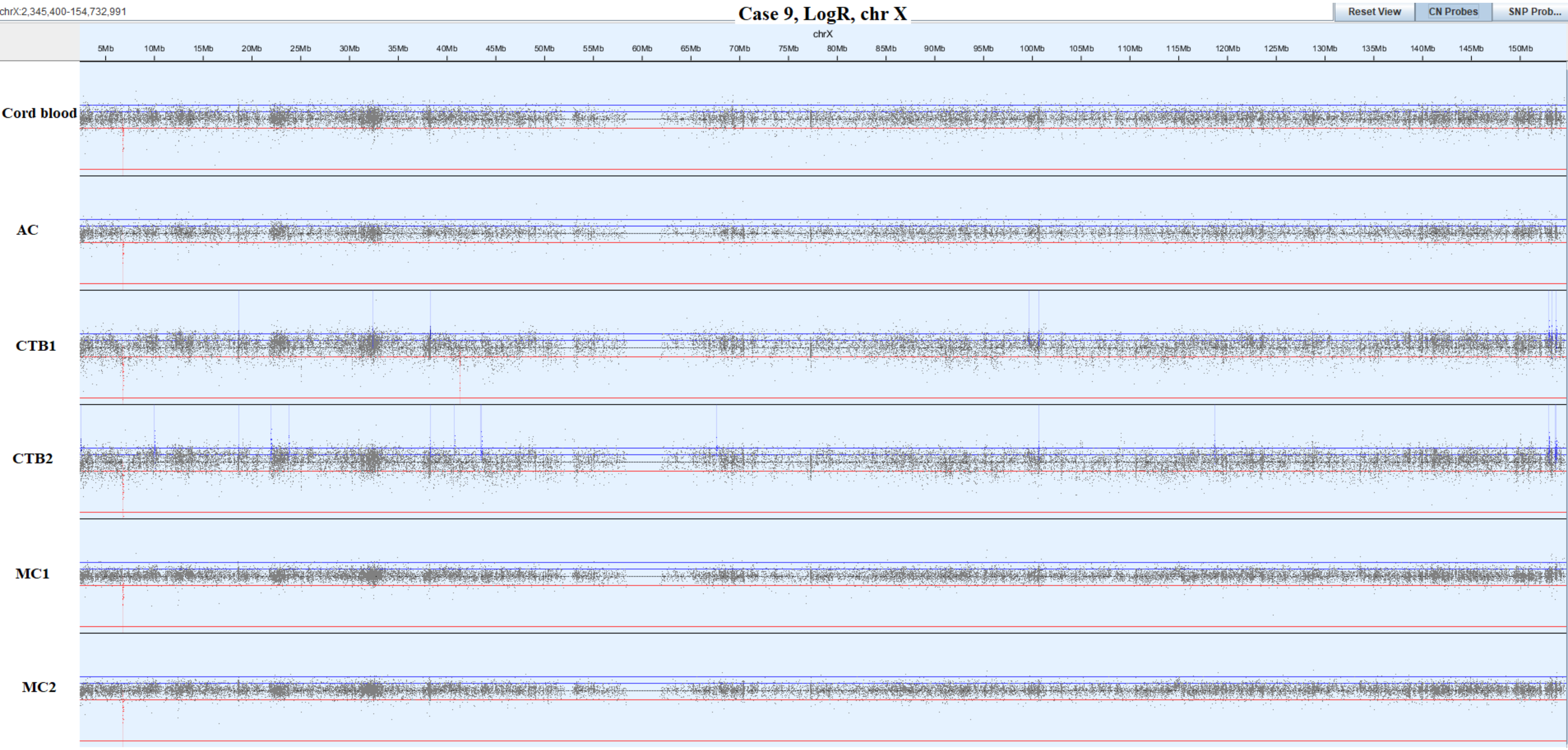
chrX:2,345,400-154,732,991
Case 9, LogR, chr X
Reset View
CN Probes
SNP Prob...
chrX
5Mb
10Mb
15Mb
20Mb
25Mb
30Mb
35Mb
40Mb
45Mb
50Mb
55Mb
60Mb
65Mb
70Mb
75Mb
80Mb
85Mb
90Mb
95Mb
100Mb
105Mb
110Mb
115Mb
120Mb
125Mb
130Mb
135Mb
140Mb
145Mb
150Mb
Cord blood
AC
CTB1
CTB2
MC1
MC2

Case 9, BAF, chr X

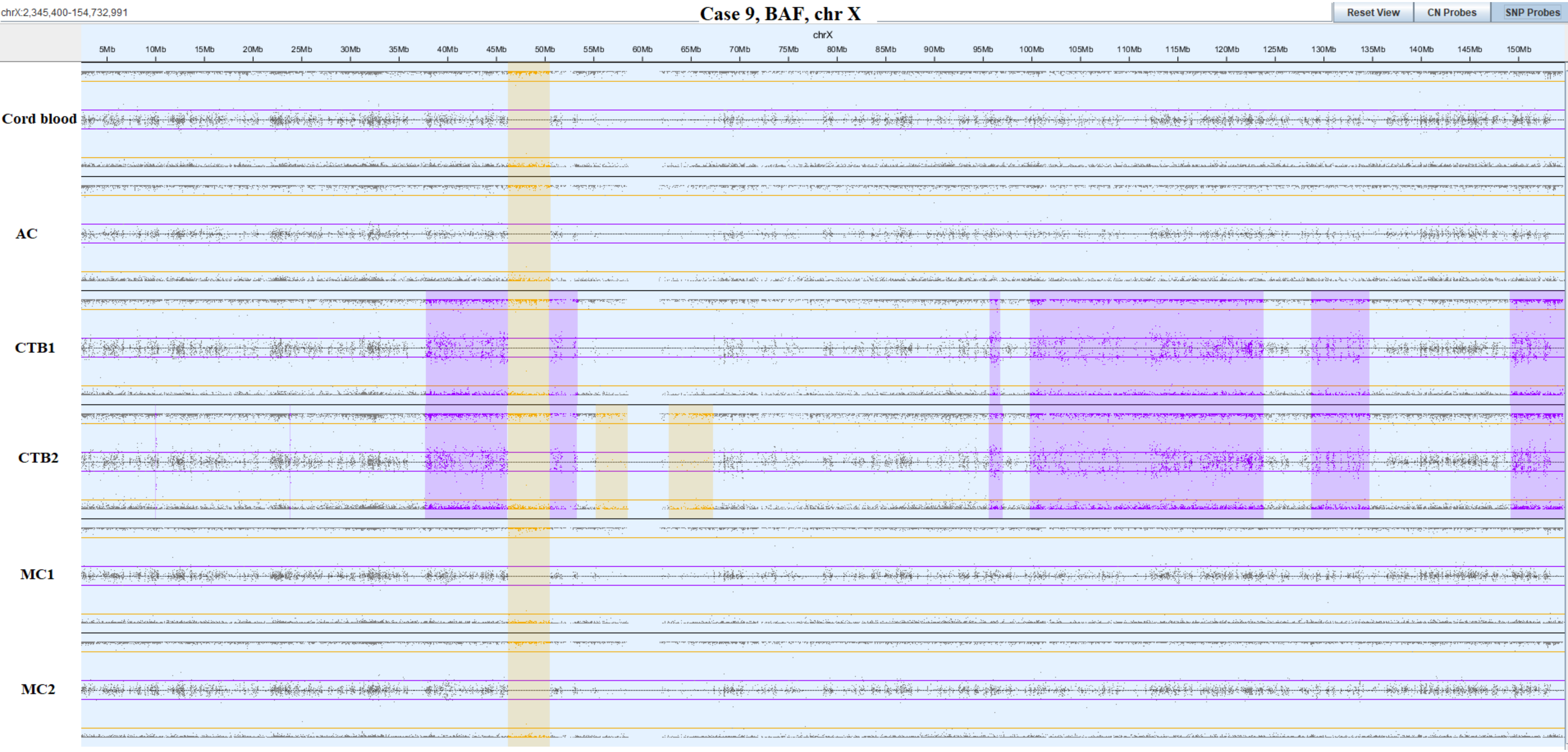

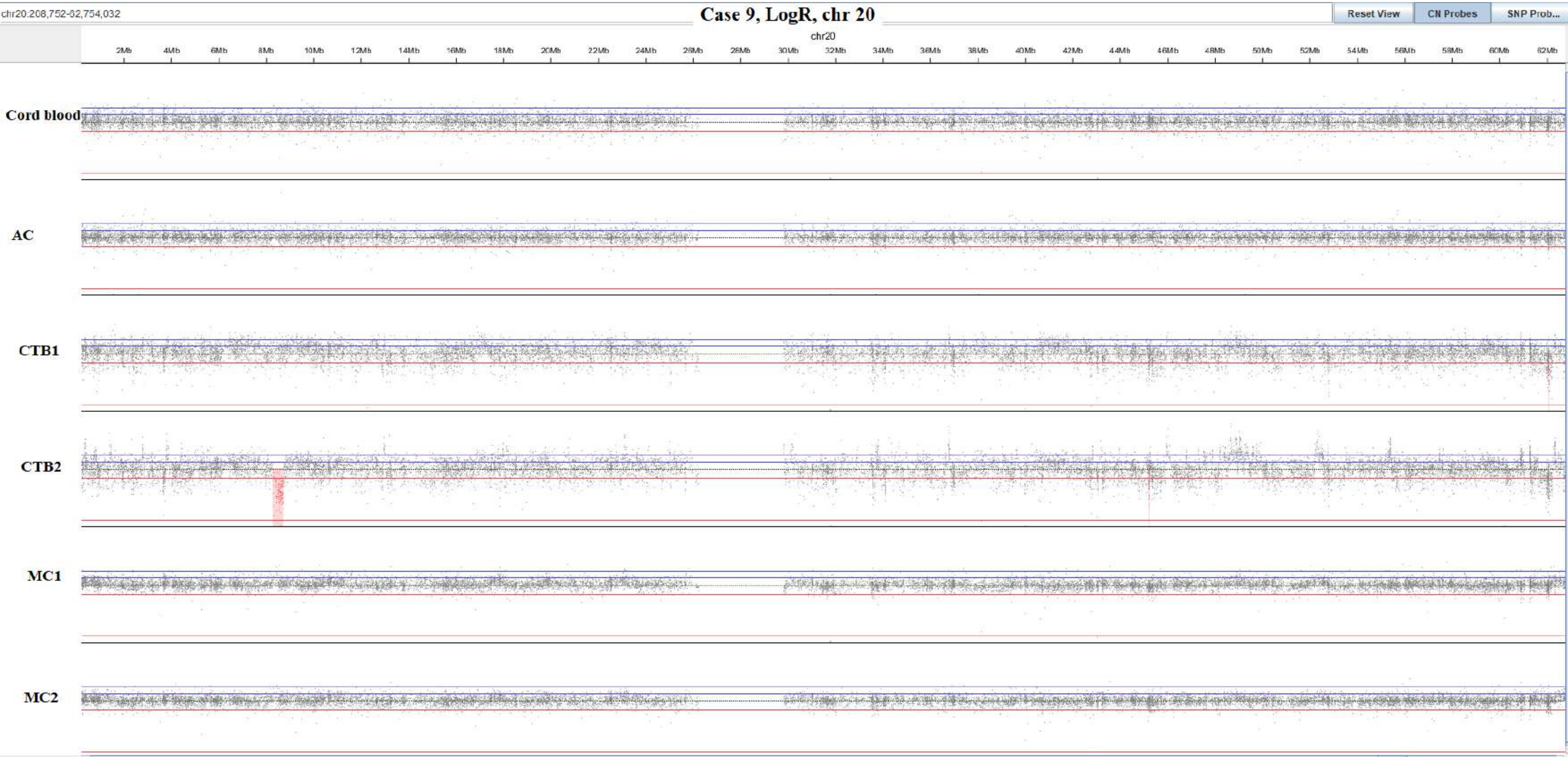

chr20:208,752-62,754,032
Case 9, LogR, chr 20
Reset View
CN Probes
SNP Prob...
chr20
2Mb
4Mb
6Mb
8Mb
10Mb
12Mb
14Mb
16Mb
18Mb
20Mb
22Mb
24Mb
26Mb
28Mb
30Mb
32Mb
34Mb
36Mb
38Mb
40Mb
42Mb
44Mb
46Mb
48Mb
50Mb
52Mb
54Mb
56Mb
58Mb
60Mb
62Mb
Cord blood
AC
CTB1
CTB2
MC1
MC2

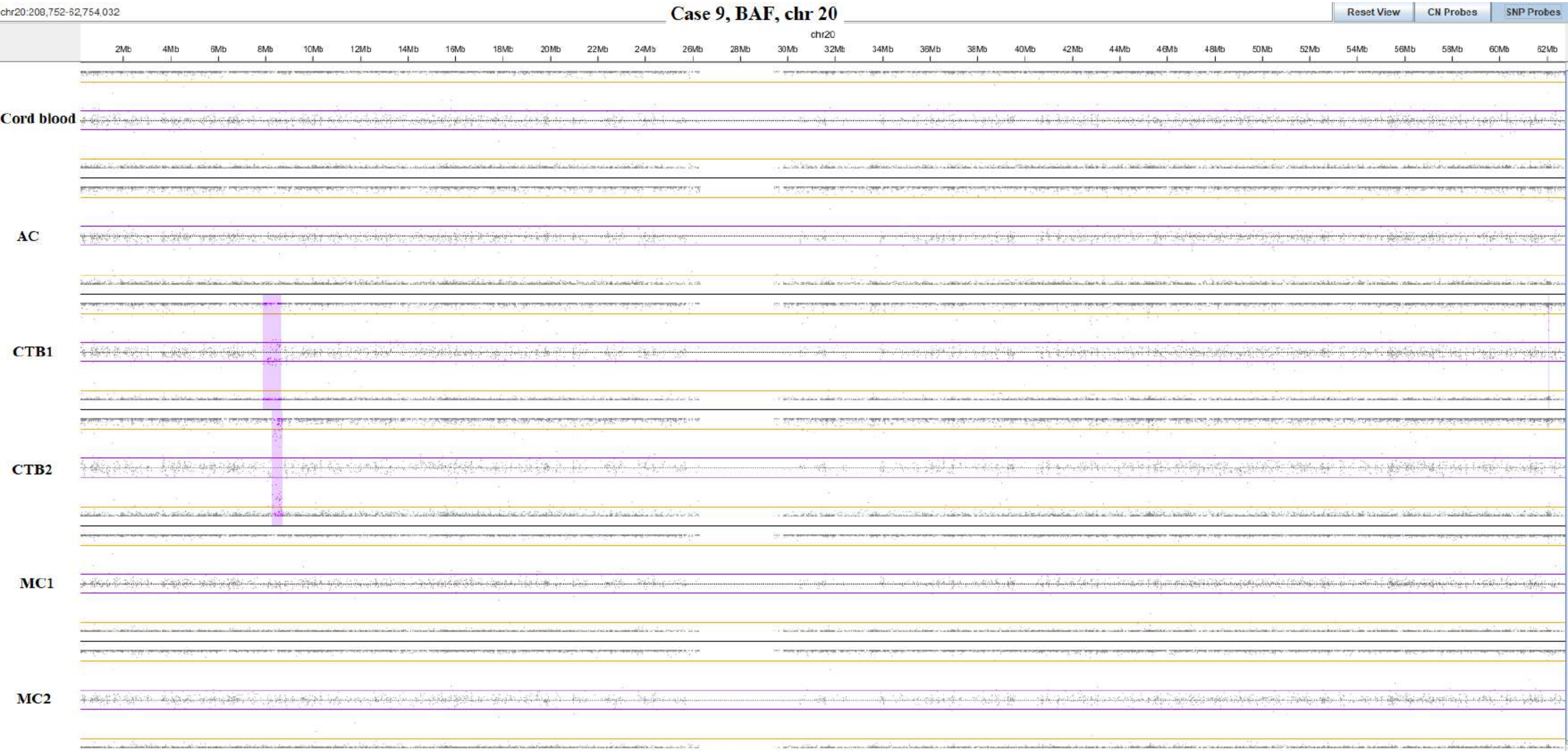

chr20:208,752-62,754,032
Case 9, BAF, chr 20
Reset View
CN Probes
SNP Probes
chr20
2Mb
4Mb
6Mb
8Mb
10Mb
12Mb
14Mb
16Mb
18Mb
20Mb
22Mb
24Mb
26Mb
28Mb
30Mb
32Mb
34Mb
36Mb
38Mb
40Mb
42Mb
44Mb
46Mb
48Mb
50Mb
52Mb
54Mb
56Mb
58Mb
60Mb
62Mb
Cord blood
AC
CTB1
CTB2
MC1
MC2
