# Supplementary material for: Studies of post-partum placentas provide insights into the origin of structural chromosomal aberrations
Source: Hum Reprod. 2025 Dec 3;41(2):168–77. doi: 10.1093/humrep/deaf235 (PMC12864147; doi:10.1093/humrep/deaf235)
Supplement: deaf235_Supplementary_Table_1 [file deaf235_supplementary_table_1.docx]

| Case no^a^ | NIPT | AF | Cord blood | Umb. chord | ppCVS | | Origin |
| --- | --- | --- | --- | --- | --- | --- | --- |
| 101  "Case 4" | Terminal loss-gain 9p | arr 9p24.3p24.1(133,828-7,497,932)x2 hmz mat  Targeted WES showed no homozygous pathogenic variants of maternal origin | - | - | CTB:  MC: | 1: arr 9p24.3p24.1(133,828-7,478,962)x1, p24.1p23(7,478,963-10,548,301)x1[0.80],p23(10,548,302-11,233,646)x3  2: arr 9p24.3p24.1(133,828-9,646,116)x1, 9p23p22.3(9,701,354-15,084,167)x3~4, mos ROH on 11p  3: arr 9p24.3p24.1(133,828-9,646,116)x1, p23p22.3(9,701,34-15,076,859)x3~4  4: arr 9p24.3p24.1(185,216-11,222,955)x1, p23(11,778,964-13,200,414)x1[0.4]  1: arr 9p24.3p24.1(133,828-7,497,932)x1  2: arr 9p24.3p24.1(133,828-7,497,932)x1[0.25], 9p24.3p24.1(133,828-7,497,932)x2 hmz mat[0.75]  3: arr 9p24.3p24.1(133,828-7,497,932)x1[0.1], 9p24.3p24.1(133,828-7,497,932)x2 hmz mat[0.9]  4: arr 9p24.3p24.1(133,828-7,497,932)x1 | Meiotic  Loss/gain 9p on paternal allele  Mos ROH on 11p: n.a. |
| T_52  "Case 5" | Terminal loss 4p | arr 4p16.3(582,067-1,312,043)x1, p16.3(~1,700,000-1,900,000)x3, p16.3p16.1(1,929,117-6,489,251)x1, 4p16.1(~6,550,000-7,000,000)x1 | - | - | CTB:  MC: | 1: arr 4p16.3(35,030-1,526,708)x1, p16.3(1,957,172-3,880,768)x1, p16.3p16.1(1,957,172-3,880,768)x1, p16.1(6,905,143-10,272,605)x3[~0.5]  2: arr 2p25.3p24.3(46,065-15,068,206)x3[~0.5-0.6], 4p16.3(35,030-1,526,708)x1~2, p16.3(1,957,172-3,880,768)x1~2, p16.3p16.1(4,238,315-6,524,054)x1~2, p16.1(~6,600,000-7,000,000)x1~2, p16.1(7,074,960-7,599,561)x2~3​  3: arr 4p16.3(35,030-1,526,708)x1, p16.3p16.1(1,957,172-3,913,083)x1, p16.1(4,238,315-6,896,337)x1  4: arr 4p16.3(35,030-1,526,708)x1, p16.3(1,957,172-3,913,083)x1, p16.3p16.1(4,238,315-6,896,337)x1  1-4: arr 4p16.3(582,067-1,312,043)x1, p16.3(~1,700,000-1,900,000)x3, p16.p16.1(1,929,117-6,489,251)x1, p16.1(~6,550,000-7,000,000)x1 | Meiotic  Loss/gain 4p on paternal allele  2p gain: n.a. (MCC) |
| T_166  "Case 6" | Interstitial gain 11q | arr 11q24.2q25(125,864,878-134,945,120)x1 | - | arr 11q24.2q25(125,864,878-134,945,120)x1 | CTB:  MC: | 1: arr 11q23.3q24.2(117,491,787-125,313,599)x3[0.2], q24.2(125,313,599-125,864,206)x1[~0.5], q24.2q25(125,864,206-134,945,120)x1[~0.8]​  2: arr 11q23.3(117,491,787-118,043,904)x3[~0.5], q23.3q24.2(118,045,846-125,313,599)x3~4, 11q24.2q25(125,335,588-134,934,063)x1  3: arr 11q23.3q24.1(117,491,787-121,519,662)x3[~0.4], q24.1q24.2(121,521,663-125,207,714)x3, q24.2q25(125,335,588-134,945,120)x1  4: arr 11q23.3(117,491,787-118,042,713)x3[~0.25], q23.3q24.1(118,045,846-121,833,868)x3~4, q24.1q24.2(121,856,320-125,074,999)x3~4, q24.2q25(125,335,588-134,945,120)x1[0.85]  1-3: arr 11q24.2q25(125,864,878-134,945,120)x1  4: arr 11q22.1q24.2(98,002,500-125,853,839)x3[0.5], q24.2q25(125,864,206-134,945,120)x1 | Meiotic  Loss on paternal allele  Gain: n.a. |
| T_459  "Case 7"^b^ | Interstitial gain 9p | arr 9p24.3(~0-200,000)x1  Karyotyping  45,XX,idic(9)[1]/46,XX [36] | - |  | CTB:  MC | 1: arr 4q22.2q35.2(93,874,380-190,937,862)x3, 9p24.3(~0-200,000)x1, p24.3p22.3(436,707-16,364,727)x3, p22.3p21.3(16,368,732-20,906,204)x3~4  2: arr 9p24.3p24.2(133,828-3,432,447)x1, p24.2p24.1(3,446,277-4,800,000)x3, p24.1(4,800,000-5,291,597)x3~4  3: arr 9p24.3(~0-350,000)x1, p24.3p13.1(396,380-39,021,035)x3[~0.9], 18p11.32(134,177-2,566,966)x2~3​  4: arr 9p24.3p24.2(133,828-3,255,143)x1, p24.2(3,270,772-4,175,400)x3, 9p24.1(~4,600,000-4,700,000)x3, 10q21.1q26.3(53,276,530-135,434,303)x3  1: arr 9p24.3 (133,828-359,395)x1[0.7], 9p24.3 (363,485-776,349)x1[0.3], 9p24.3p24.1 (781,115-5,658,775)x3[0.1], 9p24.1p23 (5,668,750-9,609,283)x3[0.8], 9p23q34.3 (9,646,116-141,068,637)x3[0.5]  2: arr 1q42.2q44(234,473,082-248,742,760)x2~3, 9p24.3 (133,828-765,828)x1[0.8], 9p24.3 (770,667-1,730,666)x1[0.6], 9p24.3q34.3 (1,731,997-141,068,637)x3[0.3]  3: arr 9p24.3 (133,828-359,395)x1, 9p24.3 (362,179-392,785)x1[0.3], 9p24.3p11.2 (396,380-45,755,225)x3, 9q13q34.3 (66,271,403-141,068,637)x3[0.6]  4: arr 9p24.3 (133,828-776,349)x1[0.6], 9p24.3q34.3 (786,299-141,068,637)x3[0.9] | Meiotic  Loss, gain 9p and gain 10q on paternal alleles  Gain 4q and 18p on maternal alleles |
| T_500  "Case 8" | Whole arm gain 4p  Terminal loss 11q | arr(X,1-22)x2 | - |  | CTB:  MC: | 1-3: arr 4p16.3p11(73,071-49,194,797)x3[~0.6], 11q24.2q25(127,268,774-134,945,120)x[~0.8]  1-2: arr(X,1-22)x2  3: arr(8)x3[0.25] | Mitotic  Gain and loss on paternal allele  T8 n.a. |
| T_135  "Case 9" | Whole arm gain 20p | arr(X,1-22)x2 | - |  | CTB:    MC: | 1-2: BAF changing across X  1-2: arr(X,1-22)x2 | Mitotic |

Supplementary Table S1: Detailed overview of case 4-9 with SNP-array results.

Text in green: Same aberration as seen with NIPT, text in orange: (likely) related aberration, text in red: (likely) unrelated aberration.

AF: amniotic fluid, Mos: mosaic, ROH: region of homozygosity, BAF: B-allele frequency, MCC: maternal cell contamination, WES: whole exome sequencing

^a^ For details on case 1-3, see: Van Opstal D, *Prenat Diagn* 2019;**39**: 1016-1025.

^b^ Published previously in: Donze SH, *Prenat Diagn*. 2024 Apr;44(4):401-408.
